# Phosphorescent Cyclometalated Palladium(II) and Platinum(II) Complexes Derived from Diaminocarbene Precursors

**DOI:** 10.1021/acs.inorgchem.3c03346

**Published:** 2024-03-12

**Authors:** Maria V. Kashina, Konstantin V. Luzyanin, Dmitry V. Dar’in, Stanislav I. Bezzubov, Mikhail A. Kinzhalov

**Affiliations:** † St. Petersburg University, 7−9 Universitetskaya Nab., St. Petersburg 199034, Russian Federation; ‡ Department of Chemistry, University of Liverpool, Crown Street, Liverpool L69 7ZD, United Kingdom; § Kurnakov Institute of General and Inorganic Chemistry, Russian Academy of Sciences, Leninskii Prosp. 31, Moscow 119991, Russian Federation

## Abstract

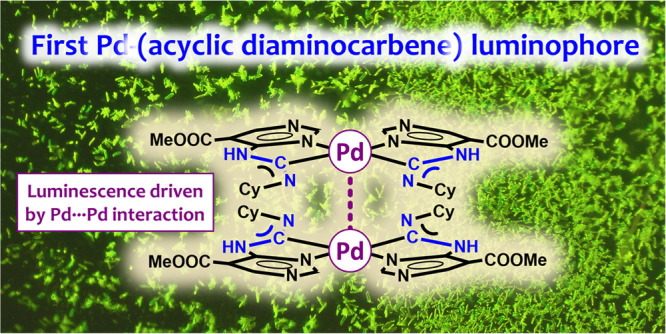

Metal-mediated self-assembly of isocyanides and methyl 4-aminopyrimidine-5-carboxylate leads to luminescent Pd^II^ and Pt^II^ complexes featuring C,N-cyclometalated acyclic diaminocarbene (ADC) ligands. The solid-state luminescent properties of these diaminocarbene derivatives are attributed to their triplet-state metal/metal-to-ligand charge-transfer (^3^MMLCT) nature, which is driven by attractive intermolecular M···M interactions further reinforced by the intramolecular π–π interactions even in the structure of the Pd compound, which is the first Pd-ADC phosphor reported.

## Abstract 2

Unique examples of Pd and Pt acyclic diaminocarbene complexes showing room-temperature aggregation-induced phosphorescence are given.

Driven by their remarkable potential as organic light-emitting diodes,^1,2^ in bioimaging,^3,4^ photocatalysis,^5^ and optical chemosensing,^6^ luminescent complexes of transition metals have attracted significant research interest. Tuning the photoemission of metal complexes requires the judicious selection of ligands, with cyclometalated aromatic species currently dominating the field for several compelling reasons.^1^ The introduction of secondary σ-donor ligands of strong ligand field, i.e., N-heterocyclic carbenes (NHCs) or acyclic diaminocarbenes (ADCs), increases the quantum efficiency and brings up the possibility of the precise tuning of emission profiles.^7^ Recent studies described cyclometalated complexes of Pt^II^ and Ir^III^ featuring ancillary NHC- and ADC-containing ligands (Figure 1A).

**Figure 1:**
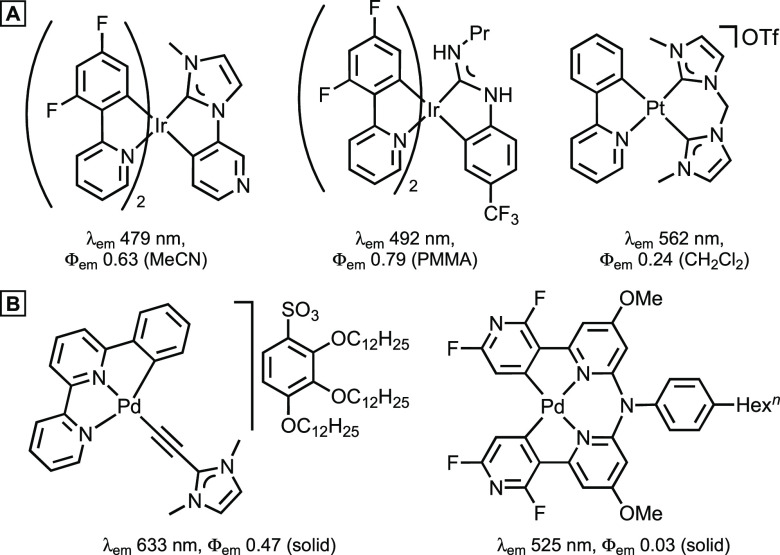
(A) Representative Pt^II^ and Ir^III^ complexes with diaminocarbenes.^8−10^ (B) Examples of Pd^II^ complexes displaying aggregation-induced phosphorescent emission.^11,12^

Whereas Pt^II^ and Ir^III^ luminescent derivatives are more explored, their Pd^II^ counterparts remain scarce, which is primarily attributed to weaker ligand-field splitting compared to that of the 5d metals arising from the less diffuse 4d orbitals of Pd^II^. Among rare examples of luminescent Pd^II^ species (Figure 1B),^11−13^ ligands of strong field and highly rigid coordination environments are prerequisites to sustain their luminescence at room temperature (RT). ADCs stand out as unique ancillary ligands showcased by a broad range of applications, and luminescent ADC complexes of Ir^III^, Pt^II^, Re^I^, and Au^I/III^ have been described.^7^

Unsurprisingly, although Pd-ADC species are widely used in transition-metal catalysis,^14,15^ to the best of our knowledge, none of them have been shown as phosphorescent (an example of a fluorescent complex has recently been reported^16^). In the present report, we reveal the preparation of a unique example of phosphorescent cyclometalated Pd^II^-ADC complexes alongside their respective Pt^II^-ADC species and elucidate their luminescent properties.

The synthetic route to new ADC complexes involves the reaction between isocyanide **1** or **2** with methyl 4-aminopyrimidine-5-carboxylate (**3**) in CHCl_3_ at RT (20–25 °C), leading to C,N-cyclometalated diaminocarbene derivatives **4** and **5** (Scheme 1, Route A). Under CHCl_3_ reflux conditions, **4** and **5** spontaneously deprotonate to the respective **6** and **7** (Route B), which were isolated in 77–79% yield. The reaction of **6** or **7** with another 1 equiv of **3** in the presence of 1,1,3,3-tetramethylguanidine as a base furnishes bis(C,N-cyclometalated) deprotonated diaminocarbene complexes **8** and **9** (Route C, 88–95% isolated yields).

**Scheme 1:**
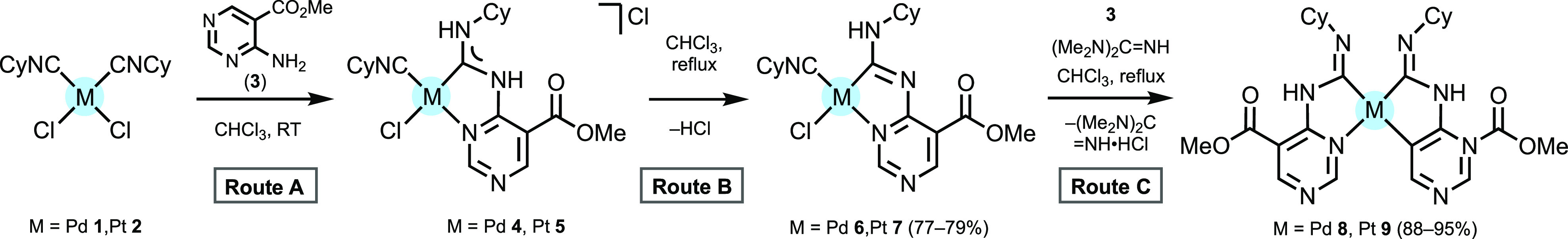
Preparation of **4**–**9**

Structural elucidation for **5**–**9**, isolated as air-stable pale-yellow to orange solids, was aided by CHN microanalyses, high-resolution positive-ion electrospray ionization mass spectromety, IR, ultraviolet/visible (UV/vis), and NMR spectroscopy, and single-crystal X-ray diffraction (XRD) for **5**–**9**. A partial conversion of **4** into **6** was observed upon evaporation of the solution; therefore, **4** was characterized only in the CDCl_3_ solution. A full description of the experimental procedures including the characterization of all compounds thus prepared can be found in the Supporting Information (SI).

Crystallization of **7** produced two crystalline forms: red crystals formed from a boiling CHCl_3_ solution (denoted as **7**^**A**^) and yellow crystals obtained via the slow evaporation of a CHCl_3_ solution at RT (denoted as **7**^**B**^). The yellow color is typical for nonaggregated C,N-cyclometalated Pt^II^ complexes, while the red is specific for solid Pt^II^ complexes featuring Pt···Pt metallophilic interactions.^17^ In the case of **6**, **8**, and **9**, only one type of crystal was obtained. The Pd^II^/Pt^II^ metal center adopts a distorted planar-square coordination geometry completed with one (**6** and **7**) or two (**9** and **10**) C,N-cyclometalated diaminocarbene ligands (Figure 2). The C–N bond distances in the diaminocarbene moiety are between typical single and double CN bonds, denoting a significant electron density delocalization in this fragment.

**Figure 2:**
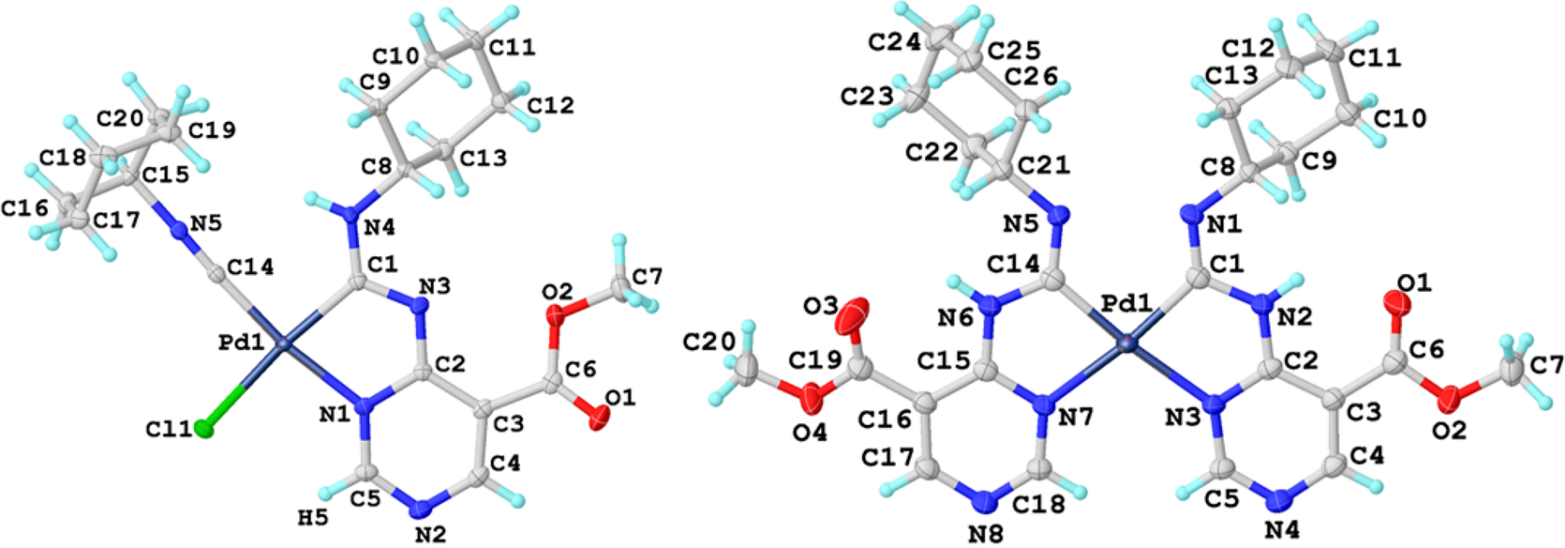
Structures of **6** (left) and **8** (right) with displacement ellipsoids at the 50% level. Crystal structures of **7**^**B**^ and **9** are similar to those of **6** and **8**, respectively, and their views are given in the SI along with relevant crystal data.

The UV/vis spectra of **5**–**9** in CH_2_Cl_2_ display high-energy intense absorption bands at 250–300 nm assigned to the transitions to ligand-centered states (π–π*), together with less intense bands at 300–400 nm (Figure S5). From the data on related complexes featuring cyclometalated diaminocarbene ligands,^18^ these bands can be associated with the transitions to mixed singlet-state ligand-to-ligand charge-transfer (^1^LL′CT) and singlet-state intraligand charge-transfer (^1^ILCT) states. It cannot, however, be ruled out that the weak band at 406 nm for the bis(cyclometalated) Pt^II^ complex is due to the direct S_1_ → T_1_ transition enabled by the large spin–orbit coupling of the Pt center. Such bands are typically not observed for the Pd^II^ counterparts due to a reduced spin–orbit coupling constant of Pd compared to Pt. Solid-state absorption spectra are different from the absorption spectra in solution; viz., they allowed low-energy bands in the spectra for **5**, **7**^**A**^, **8**, and **9** to be observed (Figure S7). The low-energy bands make Pt^II^ complex **9** look orange in color to the human eye due to transitions to metal/metal-to-ligand charge-transfer (MMLCT) states. The UV/vis spectra of **7** in the 0.03–1.2 mM concentration range were obtained (Figure S6). The shape of the UV/vis absorption spectra for the red form **7** is not influenced by the concentration within the range of 0.03–1.2 mM, and no bands after 400 nm that can be attributed to the MMLCT states are observed. This indicates that any possible aggregation has little to no impact on the ground-state behavior in the solution. UV/vis measurements of **8** and **9** with more than 0.03 mM concentration were not possible due to the low solubility of these compounds.

The deoxygenated CH_2_Cl_2_ solutions of **4**–**9** displayed negligible luminescence at RT. Upon photoexcitation at RT, crystalline **5**, **7**^**A**^, **8**, and **9** exhibited intense luminescence, with different colors ranging from green (**8**) to red (**9**) (Figure 3), while the Pd^II^ complexes **4** and **6** and Pt^II^ compound **7**^**B**^ were not emissive. All luminescent complexes show a large Stokes shift and lifetimes in the microsecond domain that clearly indicate the triplet character (phosphorescence) of the emissive state.

**Figure 3:**
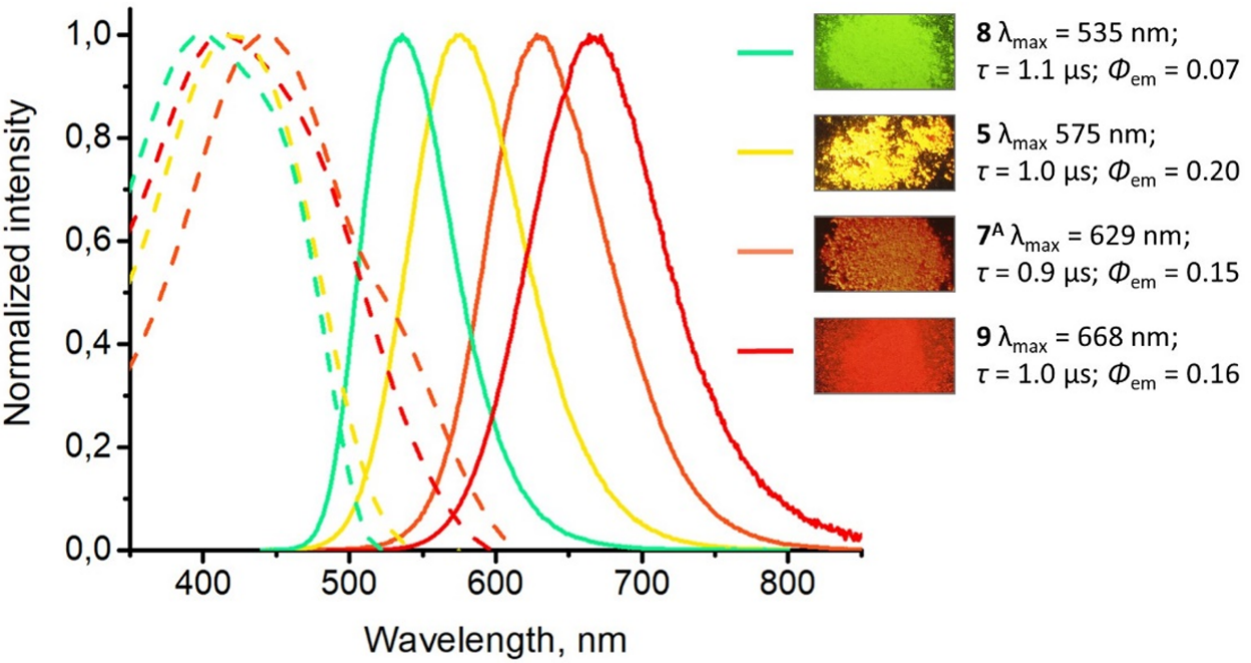
Normalized excitation (dotted lines) and emission (solid lines) spectra of **5**, **7**^**A**^, **8**, and **9** in the solid state at RT. The insets show photographs of the complexes obtained under 365 nm UV irradiation.

The nonstructured emission suggests that the excited state has a large metal orbital participation due to the formation of weak metal–metal-connected aggregates in the ground state or excimers in the excited state.^19^ This agrees with the short M···M contacts identified in **8** and **9**. For solid **8** and **9** featuring comparable metal–metal distances in their crystal structure, a red shift on going from Pd to Pt indicates an enhanced d-orbital coupling (4d_*z*^2^_ vs 5d_*z*^2^_). All crystals **5**, **7**^**A**^, **8**, and **9** show monoexponential decay with radiative rate constants (*k*_r_) as high as 10^5^ s^–1^. These high values of *k*_r_ support a triplet-state metal/metal-to-ligand charge-transfer (^3^MMLCT)-based parentage for the emitting excited state in **5**, **7**^**A**^, **8**, and **9**, which is substantially larger than the *k*_r_ value of ligand-centered excited states with typical values on the order of 10^3^ s^–1^.^2^ Grinding crystals **8** and **9** in an agate mortar induces no observable effect on their photoluminescence (Figure S14).

A phenomenon observed when a nonemissive species is induced to emit by formation of aggregates is referred to as aggregation-induced emission.^20^ Noteworthily, very few examples of Pd^II^ complexes that showed RT aggregation-induced phosphorescent emission have been reported so far (Figures 1B and S18).^11,12,21−25^ Strassert et al. described Pd^II^ complexes featuring tetradentate ligands,^12^ while Lu et al. reported pincer Pd^II^ allenylidene derivatives.^8^ Although the aforementioned reports support the role of self-assembly as an enabler of phosphorescence, the extent to which solid-state phosphorescence can be generated rationally upon chemical structure analysis is compulsive for further investigation.

In poly(methyl methacrylate) (PMMA) films, the spectrum of **8** demonstrates emission identical with that recorded in the solid state, while the spectra of Pt^II^ species **7** and **9** exhibit additional blue-shifted bands resembling the emission of a nonaggregated complex (Figures S8–S12). The emission lifetime measured for this band showed a biexponential τ_obs_, with one long-lifetime component being appreciably different from that of the main peak (Table S8). This behavior is assignable to the coexistence of both ^3^MMLCT and ^3^MLCT states. Thus, the lack of emission in solution for the Pt^II^ complexes is due to the conformational flexibility of cyclohexyl substituents, which can serve as a relaxation channel for the excited states via a nonradiative decay to the ground state.^20^ In the case of the Pd^II^ compounds, the emission of nonaggregated complexes is absent due to the population of nonradiative metal-centered states.^12^

In crystal format, compounds **8** and **9** self-organize in a head-to-tail manner, producing supramolecular dimers (Figure 4). The distances between the metal atoms in (**8**)_2_ and (**9**)_2_ [3.2312(3)–3.3379(5) Å] are comparable to the sum of Bondi’s vdW radii (0.94–1.02) but smaller than the sum of Alvarez’s (0.71–0.78) van der Waals (vdW) radii, indicating the possibility of metallophilic Pd···Pd and Pt···Pt interactions. In an attempt to understand the nature of M···M interactions, we utilized density functional theory calculations, Bader’s quantum theory of atoms-in-molecules^26^ together with a noncovalent interaction index plot^27,28^ (QTAIM/NCIplot), and analysis of the electron localization function (ELF).^29−31^

**Figure 4:**
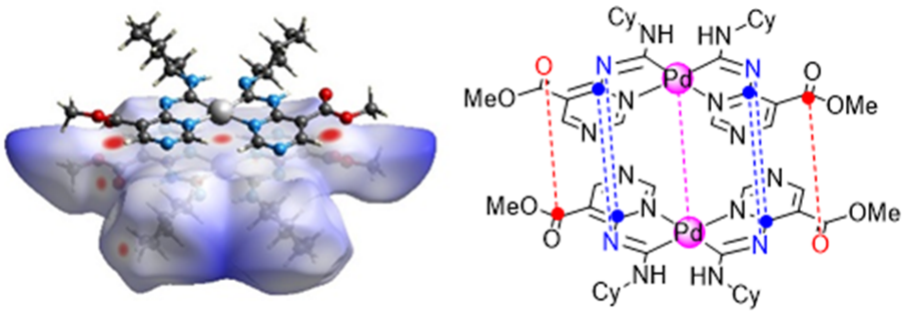
Hirshfeld surfaces for **8**. Dimeric supramolecular architecture of **8** resulting from M···M interaction (purple dotted lines), C···N (blue dotted lines), and C···O (red dotted lines) contacts. The Hirshfeld surface for **9** is similar, and its view is given in the SI.

QTAIM analysis of the computed (**8**)_2_ and (**9**)_2_ reveals that the M···M interactions are depicted by the bond critical points (BCPs) and bond paths linking the metal centers; apart from the M···M interaction, the BCPs for additional interactions (namely, C···N and C···O contacts) stabilizing the (**8**)_2_ and (**9**)_2_ assembly (Figure 5) were found. By a comparison of the QTAIM values of ρ(*r*) at the BCPs, it can be concluded that M···M is a structure-directing interaction for these supramolecular assemblies. Only a low density of ρ = 0.014–0.023 was determined, reflected in the low Mayer/Wiberg bond orders^32^ (0.16/0.18 for **8** and 0.44/0.24 for **9**) and ultimately with a weakly bonding interaction.

**Figure 5:**
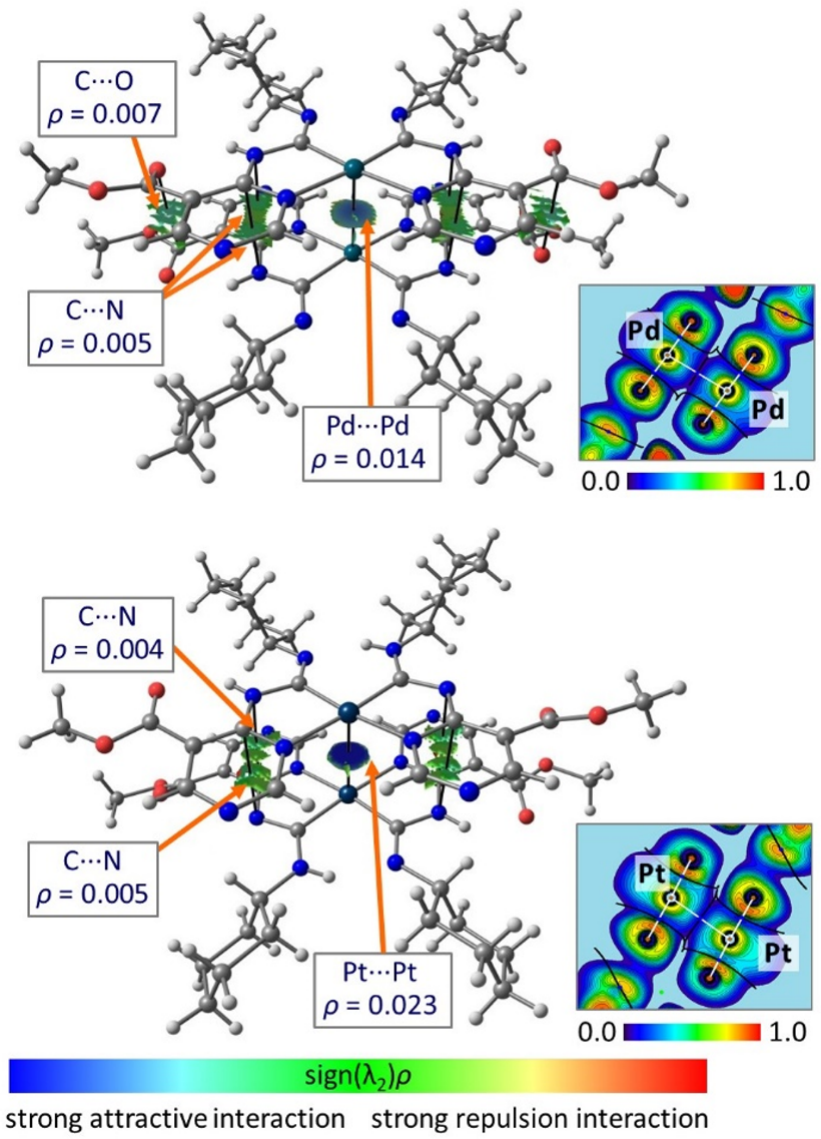
QTAIM distribution of BCPs and bond paths for the clusters (**8**)_2_ (top) and (**9**)_2_ (bottom). Only BCPs and NCIplot surfaces characterizing intermolecular interactions are represented for clarity. The insets show ELF projections for the M···M interactions in (**8**)_2_ and (**9**)_2_.

The increased ELF areas around the Pd^II^ and Pt^II^ atoms near the bond paths connecting metal centers can be interpreted as filled d_*z*^2^_ orbitals. Low ELF values between the intermetal regions indicate the absence of covalent character of these interactions. In both cases, the 1D profiles of the electron density and electrostatic potential functions^33^ along the M···M bond paths overlap (Figure S15). This confirms the nonpolar noncovalent nature of M···M interactions similar to that observed recently for other systems with metallophilic interactions.^17,34^ Based on the experimental and theoretical findings, the Pd···Pd and Pt···Pt interactions in **8** and **9** are of an intramolecular d^8^–d^8^ metallophilic nature.

In summary, we have reported a high-yielding synthesis of a series of Pd^II^ and Pt^II^ cyclometalated complexes featuring deprotonated ADCs. The strong ligand field and rigid chelate system of C,N-cyclometalated diaminocarbenes induces solid-state luminescence for Pt and bis(diaminocarbene) Pd derivatives ranging from the green to red spectral region. The emission is attributed to a long-lived triplet-manifold excited state with MMLCT character associated with the formation of attractive M···M interactions. These preliminary photophysical results show that the rational design of acyclic C,N-cyclometalated diaminocarbene-based ligands provides access to unique luminescent Pd^II^ derivatives. The emergence of intermolecular interactions is recognized as a driving force toward the phosphorescence of diaminocarbene complexes described in this work, and our future efforts will be dedicated to a better understanding of the relationship between the molecular construction and luminescence properties of metal aminocarbene species.

## Supporting Information Available

Experimental section, spectral, XRD, and experimental data, structural features discussion, and theoretical calculations (PDF)
