# Supplementary material for: Phosphorescent Cyclometalated Palladium(II) and Platinum(II) Complexes Derived from Diaminocarbene Precursors
Source: Inorg Chem. 2024 Mar 12;63(12):5315–9. doi: 10.1021/acs.inorgchem.3c03346 (PMC10966732; doi:10.1021/acs.inorgchem.3c03346)
Supplement: Supplementary file 1 — ic3c03346_si_001.pdf [file ic3c03346_si_001.pdf]

Supporting Information

Phosphorescent Cyclometallated Palladium(II) and Platinum(II) Complexes Derived from Diaminocarbene Precursors

Maria V. Kashina,[a] Konstantin V. Luzyanin,[b]* Dmitry V. Dar'in,[a] Stanislav I. Bezzubov,[c] and Mikhail A. Kinzhalov[a]*

[a]St Petersburg University, 7–9 Universitetskaya Nab., Saint Petersburg, 199034, Russian Federation.

[b]Department of Chemistry, University of Liverpool, Crown Street, Liverpool L69 7ZD, United Kingdom.

[c]Kurnakov Institute of General and Inorganic Chemistry, Russian Academy of Sciences, Leninskii Prosp. 31, 119991 Moscow, Russian Federation.

E-mails: m.kinzhalov@spbu.ru (MAK) and konstantin.luzyanin@liverpool.ac.uk (KVL)

Table of Content

S1. Materials and Instrumentation 3

S2. Synthesis and Characterization 4

S3. ^{1}H NMR monitoring of reaction between **1** and **3** and characterization of **4–9** 7

S4. X-ray diffraction studies 8

S5. Photophysical data 14

S6. Hirshfeld surface analysis 21

S7. Details of the theoretical calculations 23

S8. NMR, FTIR and MS spectra 38

References 51

S1. Materials and Instrumentation

Solvents, organic reagents were obtained from commercial sources and used as received. Isocyanide complexes *cis*-[$MCl_2(CNCy)_2$] (M = Pd **1**[1], Pt **2**[2]) were prepared as reported earlier. C, H, and N elemental analyses were carried out on a Euro EA 3028 HT CHNSO analyzer. Mass-spectra were acquired on Bruker micrOTOF spectrometer equipped with ESI source; a CH_2Cl_2/MeOH mixture was used as the solvent. The instrument was operated at positive ion mode using *m/z* range of 50–3000. The capillary voltage of the ion source was set at –4500 V (ESI$^+$) and the capillary exit at +(70–150) V. The nebulizer gas pressure was 0.4 bar and drying gas flow was 4.0 L/min. The most intensive peak in the isotopic pattern is reported. Infrared spectra were recorded on Bruker Tensor 27 FTIR instrument (4000–2400 cm^{-1}, resolution 2 cm^{-1}) in KBr pellets. The UV/vis absorption spectra in CH_2Cl_2 solution were recorded on a Shimadzu UV-2500 spectrophotometer in a quartz cuvette with l = 1.0 mm with the complex's concentration 0.03 mM. The luminescence and excitation spectra were recorded on a Fluorolog-3 (Horiba Jobin Yvon) instrument at RT. Quantum luminescence yields were determined on the same instrument with the help of direct measurement using an integrating sphere. NanoLED (pulse duration 1.1 ns, repetition rate 50 kHz) was used as an excitation source to carry out lifetime measurements. The ^{1}H, ^{13}C{^{1}H}, ^{195}Pt{^{1}H} NMR spectra, and ^{1}H, ^{13}C-HMQC/HSQC and ^{1}H,^{13}C-HMBC NMR correlation experiments were acquired on a Bruker Avance 400 spectrometer in $CDCl_3$ solvent at 20 °C. The ^{13}C CP/MAS NMR spectra were acquired using a double-resonance 4 mm MAS Bruker probe at a resonance frequency of 101 MHz under 14 kHz MAS. The CP contact time in all experiments was 3.5 μs with a delay between acquisitions of 1 sec and number of scans was collected 20000.

S2. Synthesis and Characterization

*Synthesis of methyl 4-aminopyrimidine-5-carboxylate (**3**)*

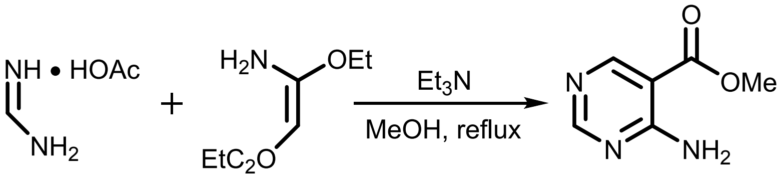

3

Scheme S1. Synthesis of methyl 4-aminopyrimidine-5-carboxylate.

A mixture of 20 g (0.126 mol) ethyl 3-amino-3-ethoxyacrylate, 31.3 g (0.3 mol) formamidine acetate, 100 mL MeOH, and 35 g (0.35 mol) Et_3N was charged to a 250 mL round bottomed flask. The mixture was stirred under reflux for 4 hours, while formamidine acetate gradually dissolved and the mixture color changed to deep yellow. The resulting solution concentrated under reduced pressure and cooled to RT. The crystals of methyl 4-aminopyrimidine-5-carboxylate (**3**) were filtered, washed with 50 mL of aqueous $NaHCO_3$ and dried on air. Yield 11.5 g, 60%.

*Synthesis of **4**.* A mixture of solid $[PdCl_2(CNCy)_2]$ (**1**, 20 mg, 0.05 mmol) and **3** (7.6 mg, 0.05 mmol) was dissolved in $CDCl_3$ (0.5 mL) and left to stand at RT for 9 days. A partial transformation of **4** into **6** was observed upon evaporation of the solution (ca. 10% of **4** was transformed **6**), so **4** was characterized only in the $CDCl_3$ solution by HR MS (ESI$^+$), 1D (1H, $^{13}C\{^1H\}$) and 2D (^1H-^1H ROESY, ^1H-^{13}C-HSQC/HMBC, ^1H-^{15}N HSQC/HMBC) NMR.

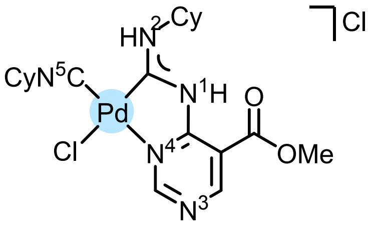

4. HR MS (ESI$^+$, *m/z*): calc. for $C_{23}H_{37}ClN_5O_2Pd^+$ 514.1044, found 514.1042 $[M – Cl]^+$. ^1H NMR ($CDCl_3$, δ): 13.94 (s, 1H, $NH_{carbene}$–Cy), 12.08 (s, 1H, $NH_{carbene}$), 9.74 (s, 1H, H), 9.33 (s, 1H, H^2), 4.22 (s, 3H, OMe), 4.07–4.14 (m, 1H, H from Cy), 3.59–3.67 (m, 1H H from Cy), 1.02–1.27 (m, 20H, H from Cy). $^{13}C\{^1H\}$ NMR ($CDCl_3$, δ): 181.47 ($NC_{carbene}N$), 163.16 (CH), 161.63, 160.25, 159.89 (CH), 121.41 (C≡N), 109.54 (C), 62.00 (OMe), 56.61 (CH from Cy), 54.51 (CH from Cy), 32.54 (CH_2 from Cy), 31.79 (CH_2 from Cy), 24.73 (CH_2 from Cy), 24.43 (CH_2 from Cy), 24.27 (CH_2 from Cy). $^{14}N\{^1H\}$ ($CDCl_3$, δ): 275 (N^3), 196 (N^4), 193 (N^2), 184 (N^5), 152 (N^1).

*Synthesis of **5**.* A mixture of solid $[PtCl_2(CNCy)_2]$ (**2**, 48.4 mg, 0.10 mmol) and **3** (15.2 mg, 0.10 mmol) was dissolved in $CHCl_3$ (5 mL) and left to stand at RT for 9 days. Then, the reaction mixture was slow evaporated in air at RT to give yellow powder of **5**. The solid was washed with three 2 mL portions of Et_2O and then dried in air at RT.

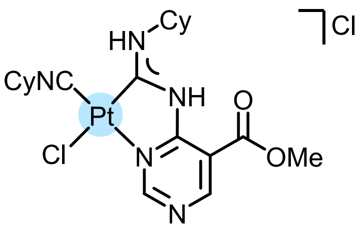

5. Yield 49 mg, 77%. Calc. for $C_{20}H_{29}Cl_2N_5O_2Pt$: C, 37.7; H, 4.6; N, 11.0. Found: C, 38.1; H, 4.9; N, 10.7. HR MS (ESI$^+$, *m/z*): calc. for $C_{23}H_{37}ClN_5O_2Pt^+$ 601.1653, found 601.1669 [M – Cl]$^+$. IR (KBr, selected bands, cm^{-1}): ν(N–H) 3432 (m); ν(C–H) 2930 (s), 2856 (s); ν(C≡N) 2251 (s); ν(C=O) 1733 (s); ν($C_{carbene}$–N) 1598 (s). ^{1}H NMR ($CDCl_3$, δ): 13.73 (s flanked by ^{195}Pt satellites, $J_{Pt,H}$ = 80 Hz, 1H, $NH_{carbene}$–Cy), 12.41 (s flanked by ^{195}Pt satellites, $J_{Pt,H}$ = 40 Hz, 1H, $NH_{carbene}$), 9.94 (s, 1H, H), 9.35 (s, 1H, H), 4.24 (s, 3H, OMe), 4.14–4.20 (m, 1H, H from Cy), 3.65–3.70 (m, 1H, H from Cy), 1.18–2.08 (m, 20H, H from Cy). $^{13}C\{^1H\}$ NMR ($CDCl_3$, δ): 170.18 ($C_{carbene}$), 163.14 (CH), 161.47, 160.82, 158.43 (CH), 109.14 (C), 105.07 (C≡N), 60.65 (CH from Cy), 56.60 (CH from Cy), 54.54 (OMe), 32.46 (CH_2 from Cy), 31.65 (CH_2 from Cy), 24.71 (CH_2 from Cy), 24.54 (CH_2 from Cy), 22.39 (CH_2 from Cy).

*Synthesis of **6**–**7**. Method A.* Solution of **4** or **5** (0.10 mmol) in $CHCl_3$ (5 mL) was stirred for 24 h under reflux. The mixture color was turning from colorless to pale yellow. Then, the reaction mixture was slow evaporated in air at RT to give yellow (**6**) or orange (**7**) crystals. The solids of **6**–**7** were washed with three 2 mL portions of Et_2O and then dried in air at RT. *Method B.* Method B is analogous to synthesis of **4** and **5**, but the reaction mixture was stirred for 24 h under reflux.

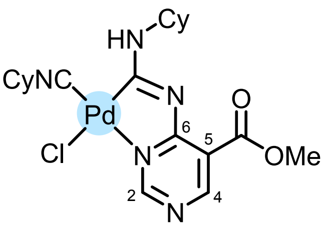

6. Yield 48 mg, 92%. Calc. for $C_{20}H_{28}ClN_5O_2Pd$: C, 46.9; H, 5.5; N, 13.7. Found: C, 46.2; H, 5.5; N, 13.2. HR MS (ESI$^+$, *m/z*): calc. for $C_{20}H_{29}ClN_5O_2Pd^+$ 514.1044, found 514.1044 [M + H]$^+$. IR (KBr, selected bands, cm^{-1}): ν(N–H) 3403 (m); ν(C–H) 2929 (s), 2854 (s); ν(C≡N) 2224 (s); ν(C=O) 1709 (s); ν($C_{carbene}$–N) 1589 (s). ^{1}H NMR ($CDCl_3$, 400.13 MHz, δ): 9.21 (s, 1H, H^3), 8.89 (s, 1H, H^2), 5.82 (d, J = 6.6 Hz, 1H, $NH_{carbene}$), 4.35–4.18 (m, 1H, H from Cy), 4.16–4.03 (m, 1H, H from Cy), 3.92 (s, 3H, OMe), 2.30–1.12 (m, 20H, H from Cy). $^{13}C\{^1H\}$ NMR ($CDCl_3$, δ): 187.25 ($C_{carbene}$), 172.01 (C^6), 164.61 (C=O), 160.79 (C^2), 159.43 (C^4), 130.05 (C≡N), 113.78 (C^5), 55.63 (CH from Cy), 53.89 (CH from Cy), 52.26 (OMe), 32.73 (CH_2 from Cy), 32.00 (CH_2 from Cy), 25.43 (CH_2 from Cy), 24.58 (CH_2 from Cy), 22.69 (CH_2 from Cy).

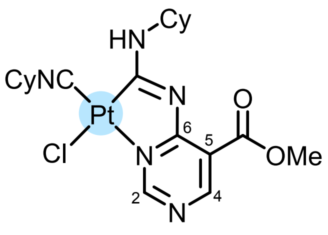

7. Yield 56 mg, 94%. Calc. for $C_{20}H_{28}N_5ClO_2Pt$: C, 40.0; H, 4.7; N, 11.7, found C, 40.1; H, 4.8; N, 11.2. HR MS (ESI$^+$, *m/z*): calc. for $C_{20}H_{29}ClN_5O_2Pt^+$ 601.1653, found 601.1663 [M + H]$^+$. IR (KBr, selected bands, cm^{-1}): ν(N–H) 3419 (m); ν(C–H) 2931 (s), 2852 (s); ν(C≡N) 2223 (s); ν(C=O) 1710 (s); ν($C_{carbene}$–N) 1596 (s). ^{1}H NMR ($CDCl_3$, 400.13 MHz, δ): 9.34 (s, 1H, H^3), 8.84 (s, 1H, H^2), 5.97 (d, J = 7.2 Hz, 1H, $NH_{carbene}$), 4.35–4.19 (m, 1H, H from Cy), 4.18–3.99 (m, 1H, H from Cy), 3.91 (s, 3H, OMe), 2.31–1.04 (m,

20H, H from Cy). $^{13}C\{^1H\}$ NMR ($CDCl_3$, 100.16 MHz, δ): 177.76 ($C_{carbene}$), 174.06 (C^6), 164.65 (C=O), 160.61 (C^2), 158.12 (C^4), 113.26 (C^5), 55.72 (CH from Cy), 53.01 (CH from Cy), 52.25 (OMe), 32.75 (CH_2 from Cy), 32.12 (CH_2 from Cy), 25.46 (CH_2 from Cy), 24.61 (CH_2 from Cy), 22.75 (CH_2 from Cy); the *C*N resonances were not detected even at high acquisition time. $^{195}Pt\{^1H\}$ NMR ($CDCl_3$, 86.02 MHz, δ): –3880.

Synthesis of ***8*****–*****9****. Method A.* A mixture of solid [$MCl_2(CNR)_2$] (**1**–**2**, 0.10 mmol) and **3** (0.20 mmol) with 1,1,3,3-tetramethylguanidine (0.5 mmol) in $CHCl_3$ (5 mL) was stirred overnight (10 h) under reflux to give yellow or orange solution over the greenish yellow (**9**) or reddish orange (**10**) precipitate. These precipitates of **9**–**10** were separated by centrifugation, washed with two 2 mL portions of Et_2O and dried in air at RT. *Method B.* Method B is analogous to Method A, but solid **6** or **7** (0.10 mmol) were used instead of [$MCl_2(CNR)_2$] and a different quantity of **3** (0.10 mmol) was used.

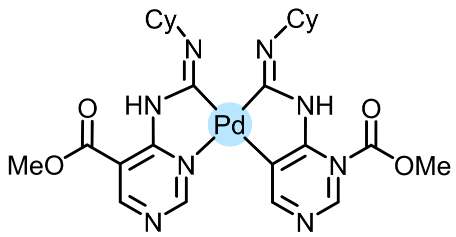

8. Yield 60 mg, 95%. Calc. for $C_{26}H_{34}N_8O_4Pd$: C, 49.6; H, 5.4; N, 17.8, found C, 49.1; H, 5.7; N, 17.6. HR MS (ESI$^+$, *m/z*): calc. for $C_{26}H_{35}N_8O_4Pd^+$ 629.1811, found 629.1813 [M + H]$^+$. IR (KBr, selected bands, cm^{-1}): ν(C–H) 2925, 2853 (m), ν(C=O) 1735 (s), 1700 (s), ν($C_{carbene}$–N) 1596 (s). ^{13}C NMR (101 MHz, CP/MAS, δ): 187.4 ($NC_{carbene}N$), 167.9 (CO), 170.5, 164.3, 161.6, 160.8, 113.9, 112.5, 108.3, 53.4, 52.1, 58.4, 54.6, 34.9, 32.6, 26.6.

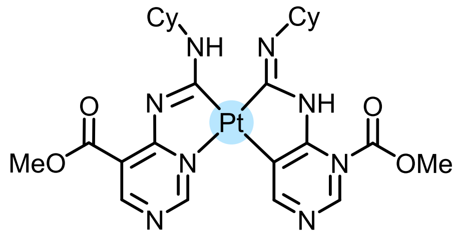

9. Yield 63 mg, 88%. Calc. for $C_{26}H_{34}N_8O_4Pt$: C, 43.5; H, 4.8; N, 15.6, found C, 43.0; H, 4.8; N, 15.3. HR MS (ESI$^+$, *m/z*): calc. for $C_{26}H_{35}N_8O_4Pt^+$ 718.2424, found 718.2436 [M + H]$^+$. IR (KBr, selected bands, cm^{-1}): ν(C–H) 2925, 2852 (m), ν(C=O) 1733 (s), 1701 (s), ν($C_{carbene}$–N) 1593 (s). ^{13}C NMR (101 MHz, CP/MAS, δ): 176.5 ($NC_{carbene}N$), 167.4, 172.3, 164.2, 161.4, 151.2, 113.9, 113.0, 108.0, 53.2, 52.1, 57.7, 34.06, 26.4.

*S3. ^{1}H NMR monitoring of reaction between **1** and **3** and characterization of **4–9***

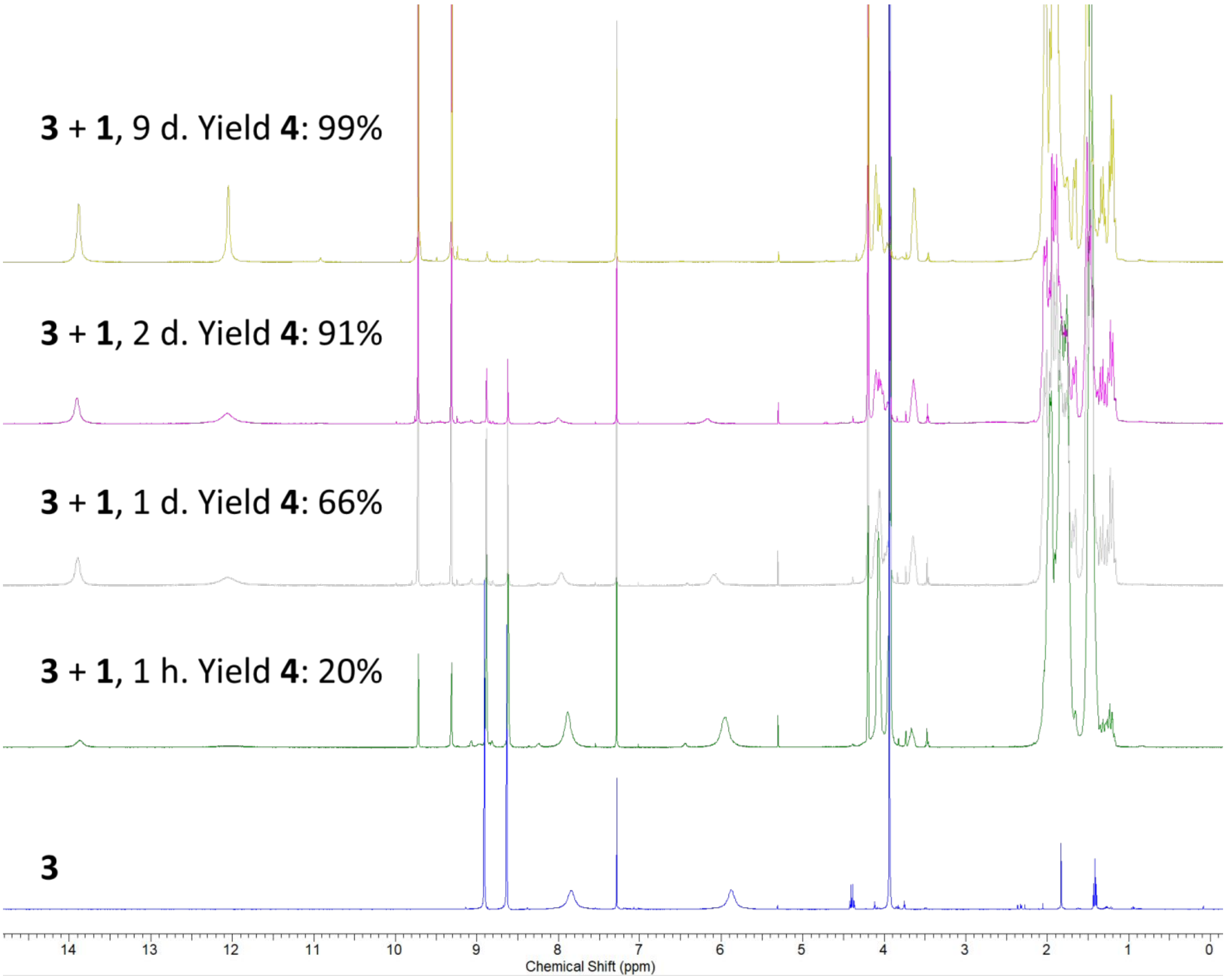

Figure S1. ^{1}H NMR monitoring of reaction between **1** and **3** in $CDCl_3$ at 25 °C.

The FTIR spectra of **5**–**9** demonstrate *ν*($C_{carbene}$–N) bands at 1596–1657 cm^{-1} indicating formation of the carbene moiety. A strong *ν*(C≡N) stretch band at *ca*. 2197–2223 cm^{-1} appeared due to the unreacted isocyanide ligand presenting in **5**–**7**, while the absence of *ν*(C≡N) bands for **8**–**9** indicating its transformation or elimination. The ^{1}H NMR spectra show the signals of $C_{carbene}$NH protons in aminocarbene-like species **6**, **7** at 5.82, 5.97 ppm, while the protons in diaminocarbene compounds **4**–**5** resonate upfield at 12.08–12.41 and 13.73–13.94 ppm due to involving in hydrogen bonds with chloride anion and C=O in ester group. The $C_{carbene}$NH in **6**, **7** is exocyclic which is detected by the cross peaks of NH signal with the signal of CH from Cy ring in ^{1}H, ^{13}C-HSQC and ^{1}H,^{13}C-HMBC. The $C_{carbene}$ atoms in **4**–**9** resonate at 176.5–187.4 ppm which is in accordance with data for other diaminocarbene Pd^{II}, Pt^{II} complexes.[1, 3-7]

S4. X-ray diffraction studies

Single crystals of **6–7** were obtained by slow evaporation of its solution in $CHCl_3$, while **8–9** were crystallized in reaction mixture. Suitable crystals were mounted on a MiTeGen tip via crystallographic oil. Data were collected using a Rigaku XtaLAB Synergy, Single source at home/near, HyPix diffractometer equipped (monochromated Cu*Kα* radiation, λ = 1.54184 Å) at 100(0) K. In each case, the structure was solved with a ShelXT[8] structure solution program using Intrinsic Phasing and refined with a ShelXL[8] refinement package incorporated in the OLEX[2] program package[9] using Least Squares minimization. Empirical absorption correction was applied in CrysAlisPro[10] program complex using spherical harmonics, implemented in SCALE3 ABSPACK scaling algorithm.

Table S1. Crystal data and structure refinement for **6–9**.

**Identification code**	**6**	**7**	**8**	**9**
CCDC	2260954	2260931	2260932	2260928
Empirical formula	$C_{22}H_{28}ClN_5O_2Pd$	$C_{20}H_{28}ClN_5O_2Pt$	$C_{26}H_{34}N_8O_4Pd$	$C_{26}H_{34}N_8O_4Pt$
Formula weight	512.32	601.01	629.01	717.70
Temperature/K	100.01(10)	100.15	150.0	100.00(10)
Crystal system	monoclinic	monoclinic	triclinic	triclinic
Space group	$P2_1/n$	$P2_1/c$	P-1	P-1
a/Å	10.0343(2)	10.1492(3)	9.5256(4)	10.06110(10)
b/Å	21.2595(5)	21.1115(6)	10.3695(5)	11.4680(2)
c/Å	10.2841(2)	10.2269(3)	15.0104(6)	14.5004(2)
α/°	90	90	105.7560(10)	109.9180(10)
β/°	104.544(2)	103.992(3)	107.0830(10)	106.9620(10)
γ/°	90	90	97.089(2)	91.3550(10)
Volume/Å^3	2123.55(8)	2126.25(11)	1330.32(10)	1490.41(4)
Z	4	4	2	2
Radiation	MoKα (λ =0.71073)	CuKα (λ = 1.54184)	MoKα (λ = 0.71073)	CuKα (λ = 1.54184)
2Θ range for data collection/°	6.358 to 55	8.376 to 151.938	4.286 to 54.996	6.842 to 134.966
Index ranges	-13 ≤ h ≤ 13, -27 ≤ k ≤ 27, -13 ≤ l ≤ 13	-12 ≤ h ≤ 12, -26 ≤ k ≤ 17, -12 ≤ l ≤ 10	-12 ≤ h ≤ 12, -13 ≤ k ≤ 13, -19 ≤ l ≤ 19	-12 ≤ h ≤ 12, -13 ≤ k ≤ 13, -17 ≤ l ≤ 17
Goodness–of–fit on F^2	1.083	1.055	1.041	1.051
Final R indexes [I≥2σ (I)]	R1 = 0.0266, wR2 = 0.0583	R_1 = 0.0323, wR_2 = 0.0883	R_1 = 0.0396, wR_2 = 0.0902	R_1 = 0.0309, wR_2 = 0.0801

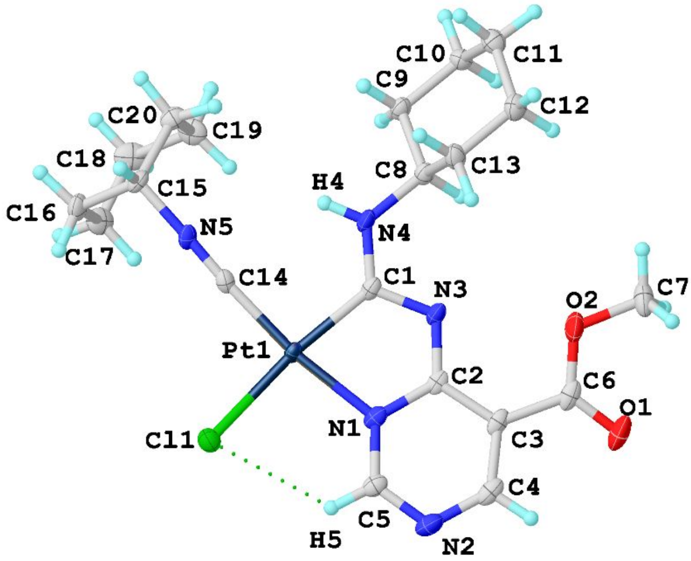

Figure S2. View of **7** with the atomic numbering schemes.

Complexes **6**–**7** exhibit a distorted planar-square geometry around the metal centers (the angles at the metal atom are in the range 80–106º). The isocyanide ligands in **6–7** are in *trans*-position to N atom from pyrimidine ring. The fragments M–C≡N–C are almost linear (**Tables S2–S3**) indicating the weak π-acceptor effect of isocyanide ligands. The bond lengths of the C≡N group (1.137(6)–1.143(3) Å) are of typical values for the CN triple bonds in palladium(II) and platinum(II) isocyanide complexes (1.135–1.177 Å).[11-16] The carbene ligands in **6**–**7** are in *cis*-position to pyrimidine fragment. The $C_{carbene}$–N bonds **6**–**7** are similar (*d*(C1–N4) = 1.292(8)–1.327(5) Å and *d*(C1–N3) = 1.341(3)–1.366(8) Å) indicating significant delocalization of electron density in diaminocarbene moiety, despite the fact that in **6**–**7** there is only an exocyclic NH group.

Table S2. Selected bond lengths and angles for **6**.

	Bond lengths, Å		Angles, °
Pd1—Cl1	2.3835(5)	N1—Pd1—Cl1	95.47(5)
Pd1—N1	2.0441(18)	C1—Pd1—Cl1	173.72(7)
Pd1—C1	1.975(2)	C1—Pd1—N1	78.31(8
Pd1—C14	1.950(2)	C14—Pd1—Cl1	92.96(6)
O1—C6	1.203(3)	C14—Pd1—N1	171.55(8)
O2—C7	1.443(3)	C14—Pd1—C1	93.27(9)
O2—C6	1.336(3)	C2—N1—Pd1	111.26(13)
N1—C2	1.385(3)	C5—N1—Pd1	128.67(16)
N1—C5	1.338(3)	N4—C1—Pd1	124.85(17)
N4—C1	1.326(3)	N3—C1—Pd1	117.0(2)
N4—C8	1.468(3)	C6—O2—C7	114.94(19)
N3—C1	1.341(3)	C5—N1—C2	119.97(19)
N3—C2	1.337(3)	C1—N4—C8	124.37(19)
N5—C14	1.143(3)	C2—N3—C1	113.59(19)
N5—C15	1.456(3)	C14—N5—C15	178.7(2)

N2—C5	1.323(3)	C5—N2—C4	113.59(19)
N2—C4	1.347(3)	N4—C1—N3	117.0(2)
		N3—C2—N1	118.61(19)
		N2—C5—N1	126.0(2)
		N2—C4—C3	124.8(2)
		O2—C6—O1	123.6(2)

Table S3. Selected bond lengths and angles for **7**.

	Bond lengths, Å		Angles, °
Pt1—Cl1	2.3755(10)	N1—Pt1—Cl1	95.05(11)
Pt1—N1	2.040(3)	C1—Pt1—Cl1	173.21(12)
Pt1—C1	1.959(4)	C1—Pt1—N1	78.17(15)
Pt1—C14	1.922(4)	C14—Pt1—Cl1	92.81(12)
O1—C6	1.211(6)	C14—Pt1—N1	172.14(16)
O2—C7	1.452(6)	C14—Pt1—C1	93.97(17)
O2—C6	1.333(6)	C2—N1—Pt1	111.8(3)
N1—C2	1.385(6)	C5—N1—Pt1	128.0(3)
N1—C5	1.339(5)	N4—C1—Pt1	125.5(3)
N4—C1	1.327(5)	N3—C1—Pt1	118.9(3)
N4—C8	1.460(5)	C6—O2—C7	115.4(4)
N3—C1	1.361(5)	C5—N1—C2	120.1(4)
N3—C2	1.337(5)	C1—N4—C8	125.2(3)
N5—C14	1.137(6)	C2—N3—C1	112.4(3)
N5—C15	1.452(5)	C14—N5—C15	178.2(4)
N2—C5	1.317(6)	C5—N2—C4	115.4(4)
N2—C4	1.349(6)	N4—C1—N3	115.6(4)
		N3—C2—N1	118.6(3)
		N2—C5—N1	125.7(4)
		N2—C4—C3	124.4(4)
		O2—C6—O1	123.7(4)

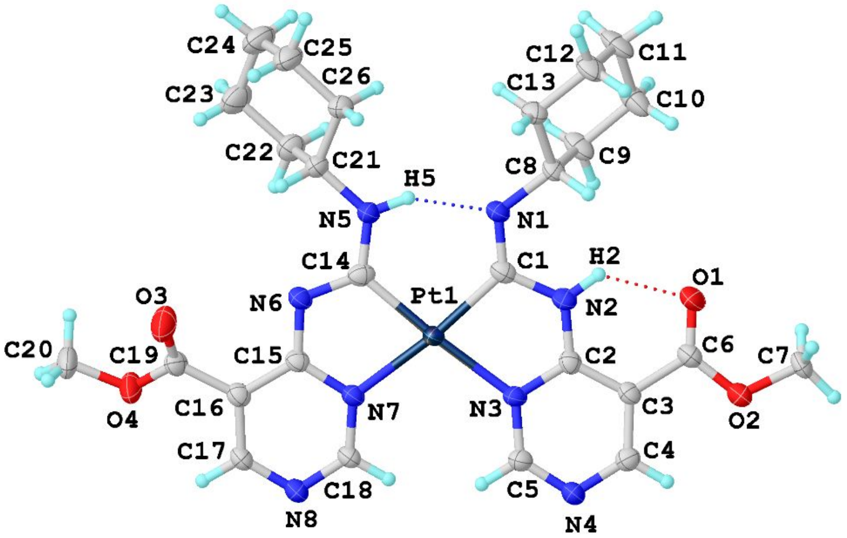

Figure S3. View of **9** with the atomic numbering schemes.

The *bis*diaminocarbene compounds **8–9** also demonstrate a distorted planar-square geometry around the metal centers (the angles at the metal atom are in the range 80–100º). The diaminocarbene moieties and pyrimidine ring form two chelating *C*,*N*-ligands with planar bicyclic framework. While the *C*,*N*-cyclometallated in structure **8** are almost mirror symmetrical, the structure of **9** possess a different arrangement of NH groups. The N*C1*N and N*C14*N fragments with endocyclic NH groups in **8** obtain amidine character and are aminocarbene-type fragments: the bond lengths *d*(C1–N2) = 1.393(4) Å and *d*(C14–N6) = 1.377(4) Å are close to the single C–N bonds, while the (C1–N1) = 1.289(4) Å and *d*(C14–N5) = 1.299(4) Å are closer to the double. The N*C1*N with endocyclic NH group in **9** are also aminocarbene-type moieties (*d*(C1–N1) = 1.292(6) Å and *d*(C1–N2) = 1.466(5) Å), while the N*C14*N with exocyclic NH is close to classical diaminocarbene fragment as $C_{carbene}$–N bonds are similar (*d*(C14–N5) = 1.314(6) Å and *d*(C14–N6) = 1.370(6) Å).

Table S4. Selected bond lengths and angles for **8**.

	Bond lengths, Å		Angles, °
Pd1—C1	1.987(3)	N7—Pd1—N3	106.42(9)
Pd1—C14	1.990(3)	C1—Pd1—N3	79.43(11)
Pd1—N3	2.147(2)	C14—Pd1—N7	78.80(11)
Pd1—N7	2.132(2)	C14—Pd1—C1	95.29(12)
C1—N1	1.289(4)	C1—Pd1—N3	79.43(11)
C1—N2	1.393(4)	C1—Pd1—N7	173.76(11)
C2—N2	1.341(4)	C6—O2—C7	116.5(3)
C2—N3	1.361(4)	C2—N3—Pd1	110.37(18)
C14—N5	1.299(4)	C2—N2—C1	119.1(3)
C14—N6	1.377(4)	C15—N7—Pd1	110.31(18)
C15—N6	1.337(4)	C15—N6—C14	117.5(3)
C15—N7	1.370(4)	C14—N5—C21	124.2(3)
N7—C18	1.337(4)	C19—O4—C20	118.2(3)
N8—C17	1.353(4)	C1—N1—C8	121.4(3)
N8—C18	1.322(4)	N6—C15—N7	118.1(3)
C17—C16	1.375(4)	O1—C6—O2	122.6(3)
C16—C15	1.420(4)	N6—C14—Pd1	115.1(2)
C16—C19	1.479(4)	N5—C14—Pd1	125.6(2)
C19—O3	1.193(4)	N5—C14—N6	119.3(3)
C19—O4	1.308(4)	N2—C1—Pd1	113.6(2)
C20—O4	1.439(4)	N1—C1—Pd1	127.7(2)
N3—C5	1.335(4)	N1—C1—N2	118.7(3)
N4—C5	1.326(4)	O3—C19—O4	122.3(3)
N4—C4	1.342(4)		
C4—C3	1.378(4)		
C3—C2	1.417(4)		
C3—C6	1.474(4)		
C6—O2	1.337(4)		
C6—O1	1.212(4)		
C7—O2	1.447(4)		

Table S5. Selected bond lengths and angles for **9**.

	Bond lengths, Å		Angles, °
Pt1—C1	1.987(4)	N7—Pt1—N3	105.91(13)
Pt1—C14	1.978(4)	C1—Pt1—N3	79.61(15)
Pt1—N3	2.131(3)	C14—Pt1—N7	77.35(16)
Pt1—N7	2.115(3)	C14—Pt1—C1	97.04(18)
C1—N1	1.292(6)	C1—Pt1—N3	79.61(15)
C1—N2	1.466(5)	C1—Pt1—N7	173.85(14)
C2—N2	1.338(5)	C6—O2—C7	116.4(3)
C2—N3	1.359(5)	C2—N3—Pt1	111.5(3)
C14—N5	1.314(6)	C2—N2—C1	119.8(4)
C14—N6	1.370(6)	C15—N7—Pt1	110.3(3)
C15—N6	1.328(5)	C15—N6—C14	113.5(4)
C15—N7	1.379(5)	C14—N5—C21	125.4(4)
N7—C18	1.332(5)	C19—O4—C20	116.8(4)
N8—C17	1.345(6)	C1—N1—C8	122.5(4)
N8—C18	1.327(6)	N6—C15—N7	119.8(4)
C17—C16	1.385(6)	O1—C6—O2	125.3(4)
C16—C15	1.428(6)	N6—C14—Pt1	118.7(3)
C16—C19	1.486(6)	N5—C14—Pt1	123.7(3)
C19—O3	1.202(6)	N5—C14—N6	117.6(4)
C19—O4	1.318(5)	N2—C1—Pt1	112.6(3)
C20—O4	1.444(6)	N1—C1—Pt1	127.8(3)
N3—C5	1.343(5)	N1—C1—N2	119.6(4)
N4—C5	1.312(6)	O3—C19—O4	121.2(4)
N4—C4	1.345(6)		
C4—C3	1.383(6)		
C3—C2	1.409(6)		
C3—C6	1.488(6)		
C6—O2	1.332(6)		
C6—O1	1.196(6)		
C7—O2	1.442(5)		

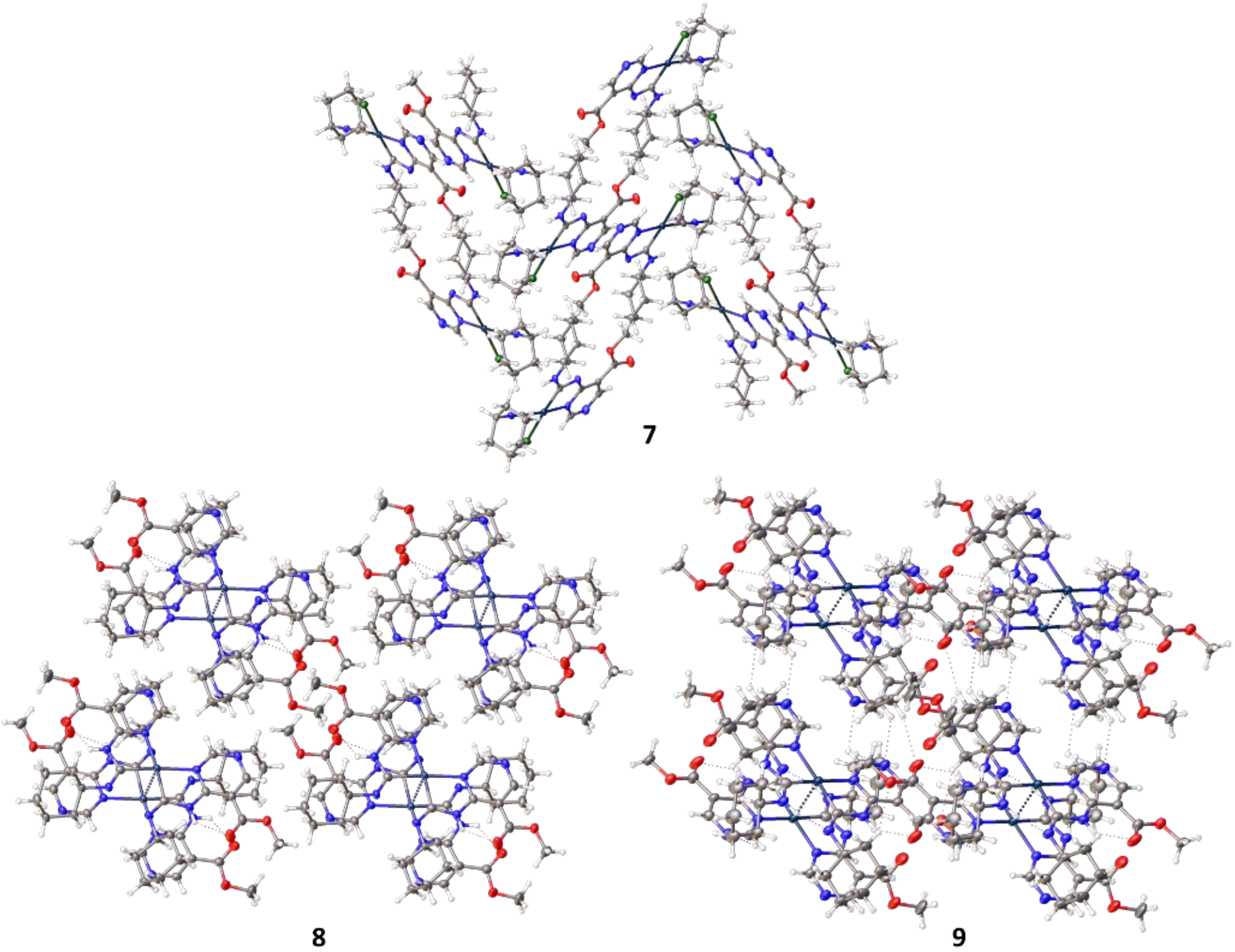

Figure S4. View of crystal packing of the **7–9** crystals along the *c* axis.

S5. Photophysical data

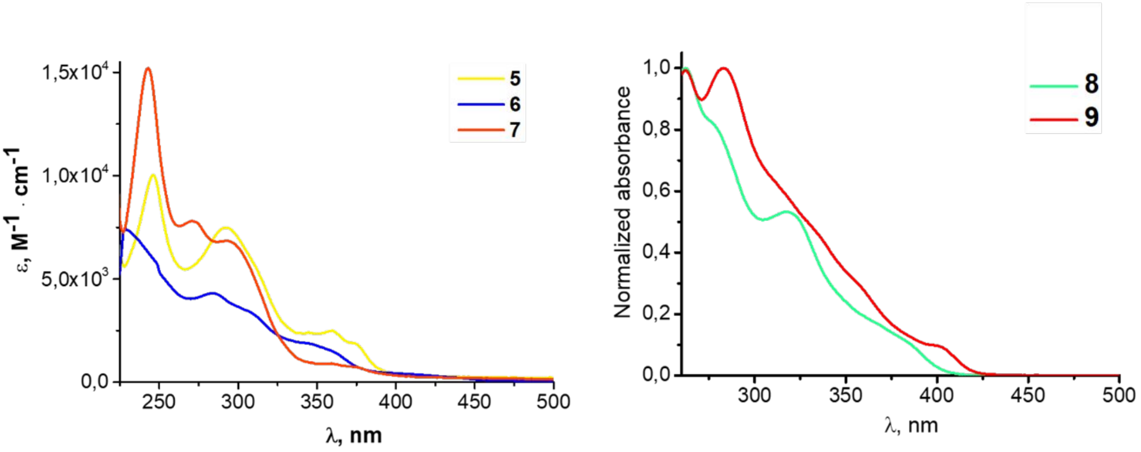

Figure S5. UV-vis absorption spectra for **5–7** (0.03 mM in CH_2Cl_2) and normalized UV-vis absorption spectra for **8–9** (saturated solution in CH_2Cl_2) at RT.

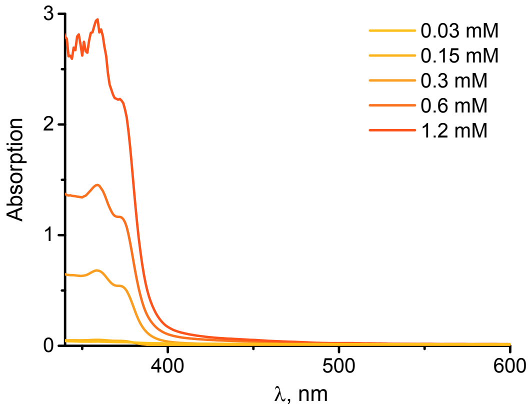

Figure S6. UV-vis absorption spectra for **7A** in CH_2Cl_2 with different concentrations of complex at RT.

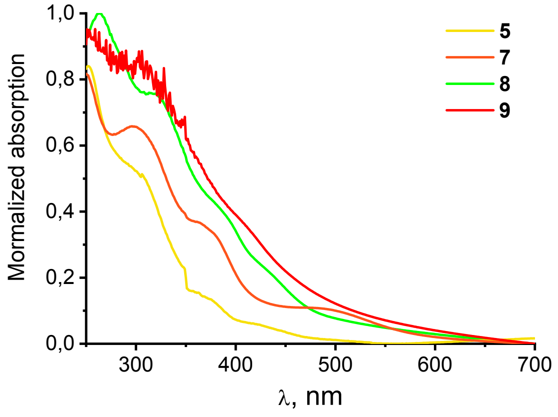

Figure S7. Normalized UV-vis absorption spectra for **5**, **7–9** in KBr pellets at RT.

Table S6. Absorption data for **5–9** in solid state and in CH_2Cl_2 solution at RT.

Complex	λ_{abs} in CH_2Cl_2, nm ($\varepsilon \times 10^{-4}$ M^{-1} cm^{-1})	λ_{abs} in KBr tablets, nm
**5**	245 (1.00), 292 (0.74), 345 (0.25), 359 (0.26), 373 (0.20)	253, 298sh, 367sh, 421sh, 493sh
**6**	230 (0.74), 285 (0.43), 309sh (0.34), 346 (0.19), 359sh (0.16), 401sh (0.05)	-
**7**[A]	243 (1.53), 273 (0.78), 294 (0.69), 359 (0.09), 374 (0.07)	251, 298, 365sh, 489sh, 618sh
**8**	262, 277sh, 318, 383sh	262, 318, 379sh, 428sh, 513sh
**9**	262, 283, 311sh, 331sh, 357sh, 401	254, 307, 348sh, 405sh, 493sh, 572sh

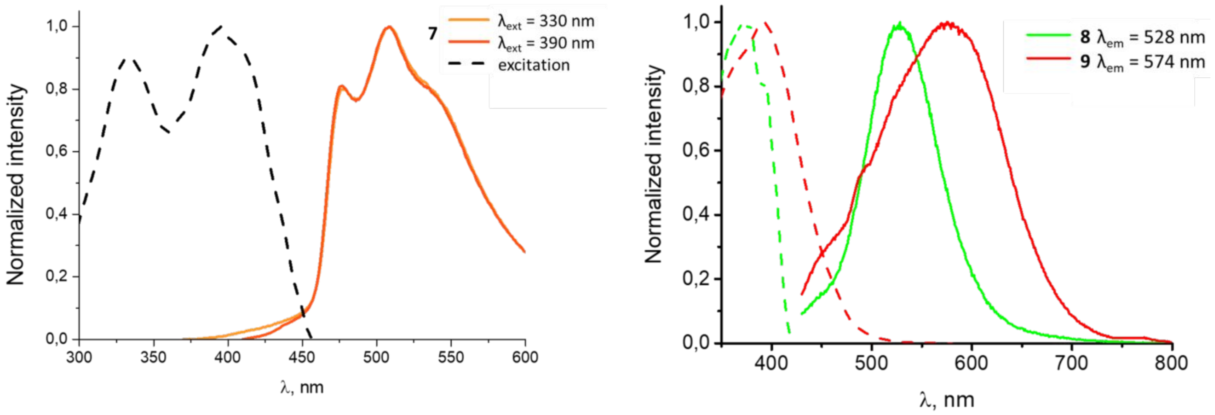

Figure S8. Normalized excitation (dotted lines) and emission (solid lines) spectra of **7–9** in PMMA at RT.

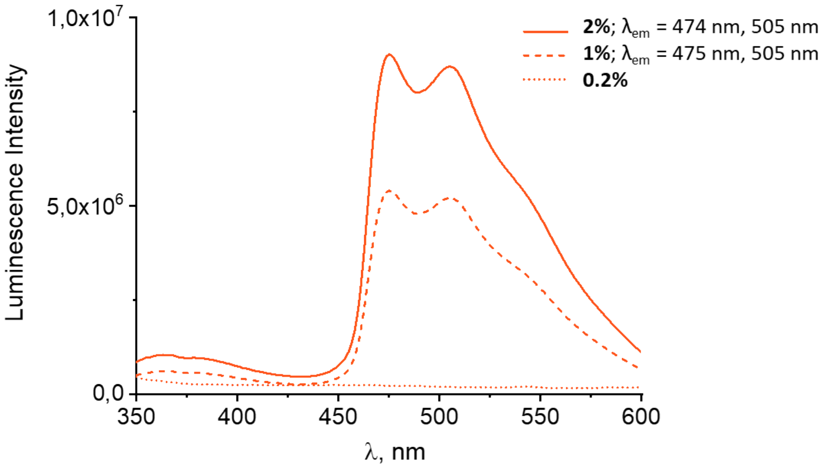

Figure S9. Emission spectra of **7** in PMMA films with 0.2%, 1% and 2% loading at RT; λ_{ext} = 330 nm.

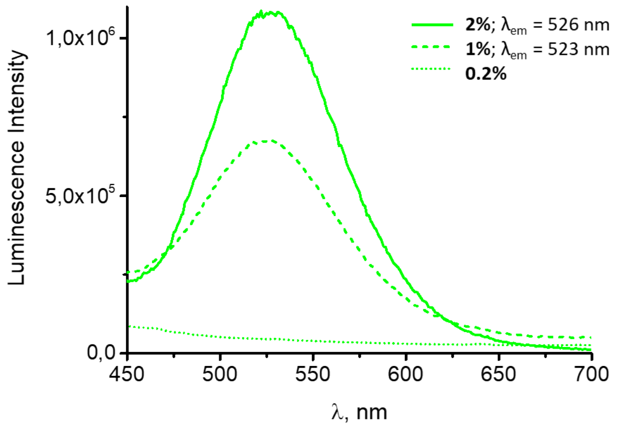

Figure S10. Emission spectra of **8** in PMMA films with 0.2%, 1% and 2% loading at RT; λ_{ext} = 390 nm.

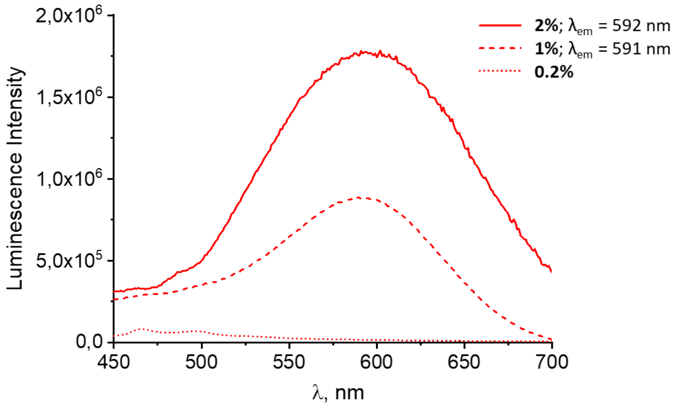

Figure S11. Emission spectra of **9** in PMMA films with 0.2%, 1% and 2% loading at RT; λ_{ext} = 410 nm.

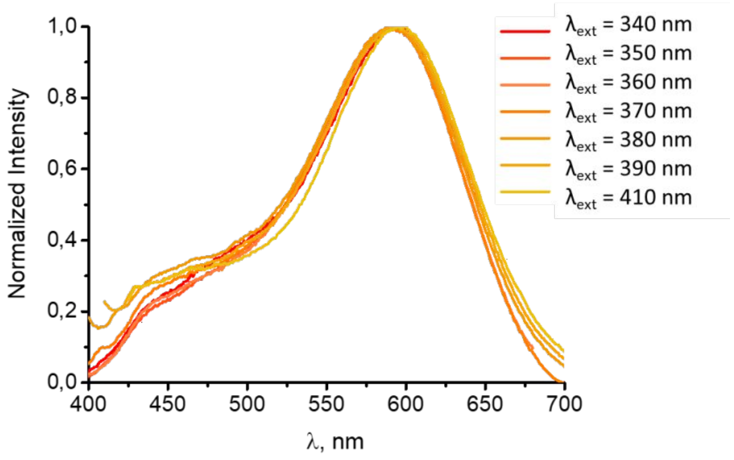

Figure S12. Emission spectra of **9** in PMMA films with 1% loading at RT after different excitation wavelength from 340 to 410 nm.

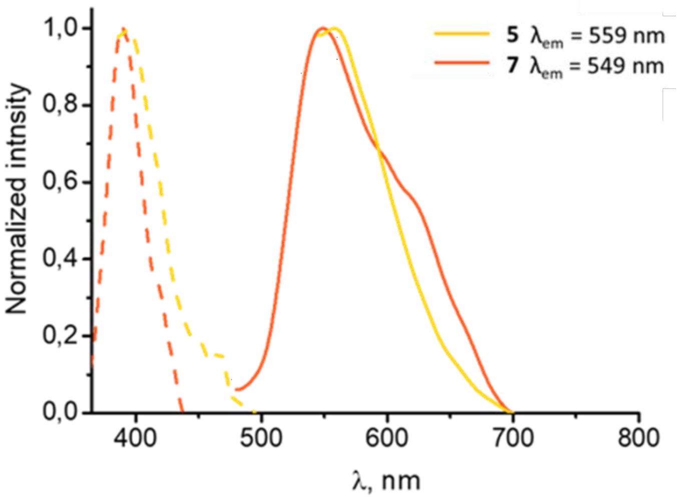

Figure S13. Normalized excitation (dotted lines) and emission (solid lines) spectra of **5** and **7** in CH_2Cl_2 at 77 K.

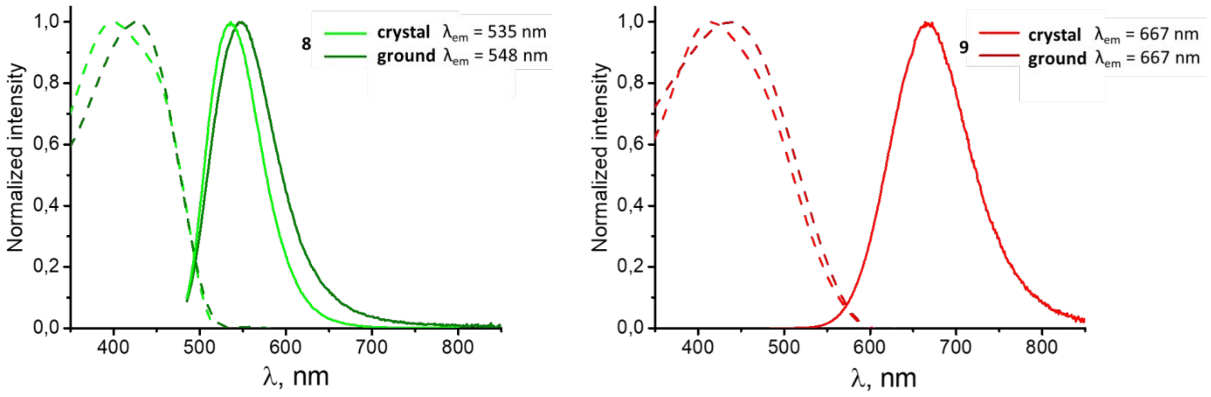

Figure S14. Normalized excitation (dotted lines) and emission (solid lines) spectra of **8** and **9** before grinding and after grinding at RT.

Table S7. Photophysical data for **5**, **7–9** in solid state at RT.

Table S6. Photophysical data for **5**, **7–9** in solid state at RT.

Complex	λ_{ems}, nm	τ, μs	Φ, %	$k_{r,}$ $10^5 \cdot s^{-1}$	$k_{nr,}$ $10^5 \cdot s^{-1}$
**5**	575	1.0 ± 0.1	20	2.0	8.0
**7**^{A}	629	0.9 ± 0.1	15	1.7	9.4
**8**	535	1.1 ± 0.1	7	0.6	8.5
**9**	668	1.0 ± 0.1	16	1.6	8.4

Table S8. Photophysical data for **7–9** in PMMA films at RT. Amplitude-weighted average lifetimes are given, as well as the different components in square brackets with relative amplitudes as percentages in parentheses.

Complex	λ_{ems}, nm	τ, μs	Φ, %
**7**[A]	477	1.1 [1.4 ± 0.2(75), 0.20 ± 0.01(25)]	
	508	1.0 [1.4 ± 0.2(64), 0.30 ± 0.01(36)]	1
	535	0.8 [1.3 ± 0.2(50), 0.30 ± 0.01(50)]	
**8**	528	0.5 [1.6 ± 0.1(21), 0.20 ± 0.02(79)]	0.5
**9**	574	0.7 [1.5 ± 0.2(33), 0.30 ± 0.03(67)]	0.5

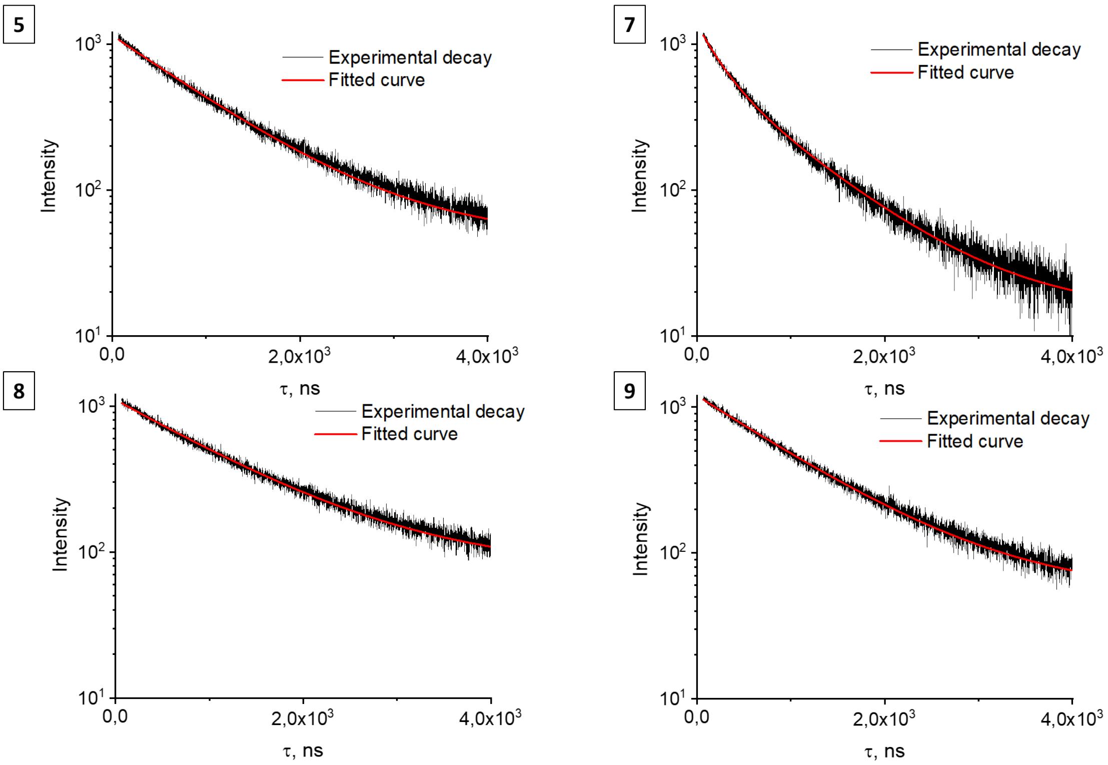

Figure S15. Time-resolved photoluminescence decays of **5**, **7–9** in solid state at RT.

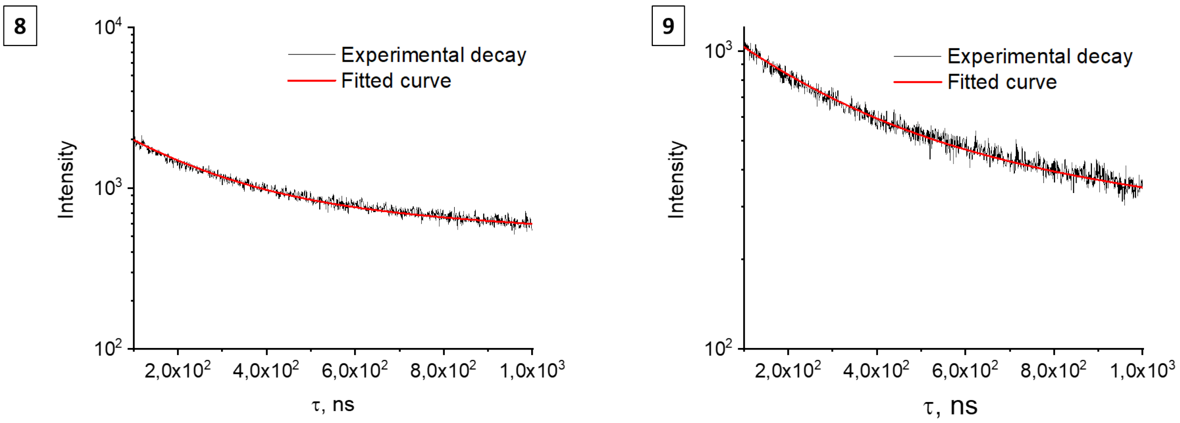

Figure S16. Time-resolved photoluminescence decays of **8–9** in PMMA films at RT.

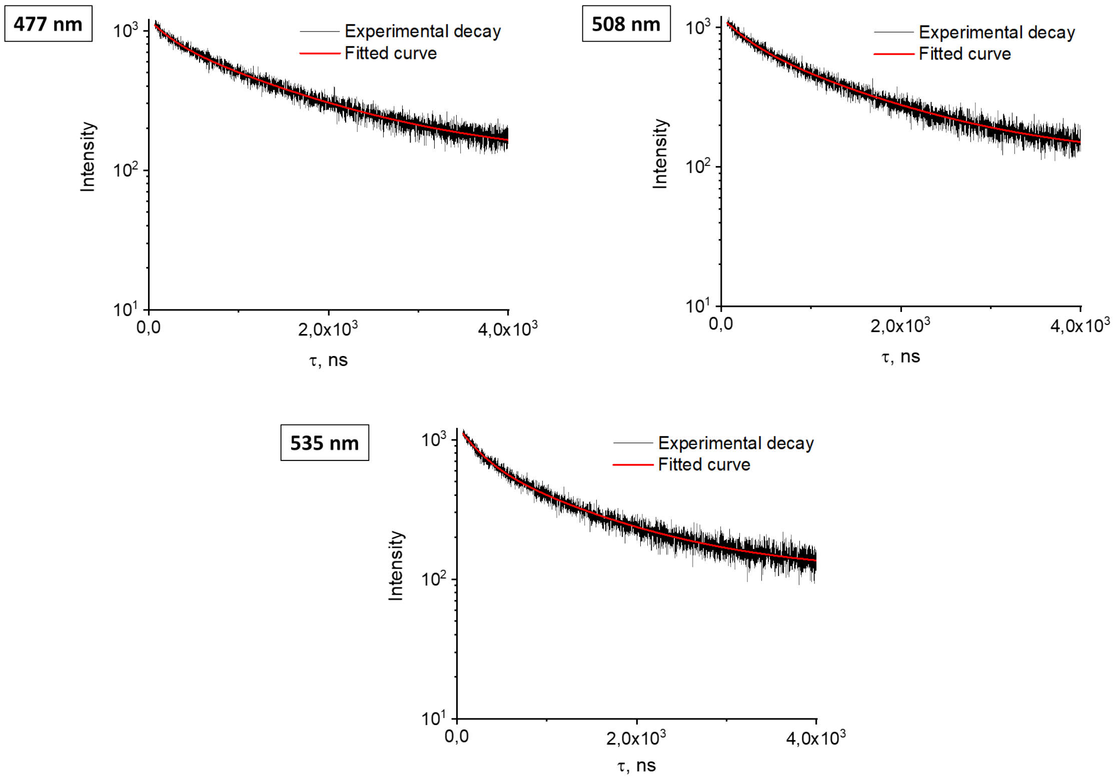

Figure S17. Time-resolved photoluminescence decays at different wavelengths of **7** in PMMA films at RT.

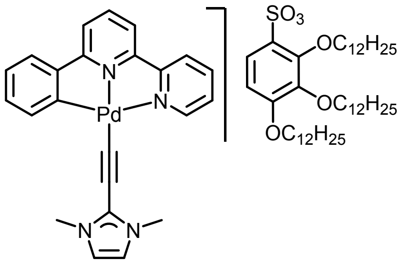

λ_{em} = 633 nm, Φ_{em} = 0.47, Ref [17]

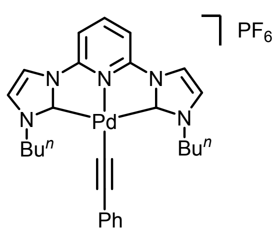

λ_{em} = 483 nm, Φ_{em} no data, Ref [18]

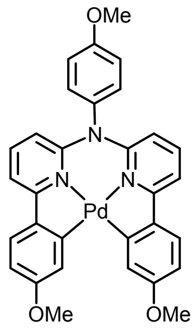

λ_{em} = 573 nm, Φ_{em} < 0.02, Ref [19]

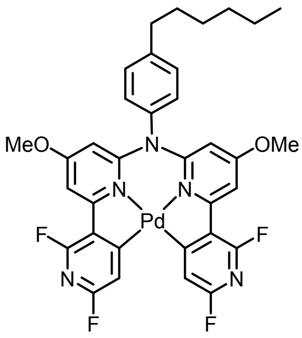

λ_{em} = 525, 568 nm, Φ_{em} = 0.03, Ref [20]

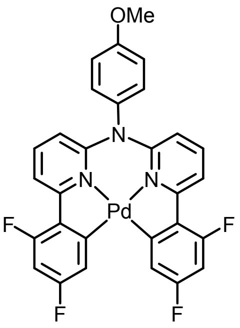

λ_{em} = 480, 560 nm, Φ_{em} < 0.02, Ref [21]

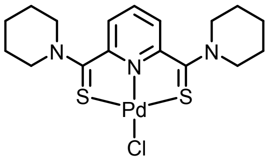

λ_{em} = 690, 715 nm, Φ_{em} no data, Ref [22]

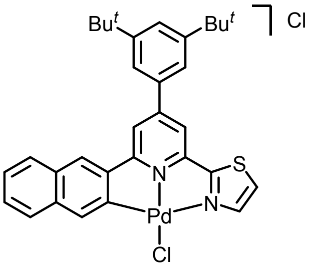

λ_{em} = 593 nm, Φ_{em} < 0.02, Ref [23]

Figure S18. Examples of Pd^{II} complexes that showed room temperature aggregation-induced phosphorescent emission.[17-23]

S6. Hirshfeld surface analysis

The Hirshfeld molecular surfaces were generated by CrystalExplorer17 program.[24-25] The normalized contact distances, d_{norm},[26] based on Bondi's van der Waals radii,[27] were mapped into the Hirshfeld surface (**Table S9** and **Figures S19**–**S20**). In the color scale, negative values of d_{norm} are visualized by the red color indicating contacts shorter than the sum of van der Waals radii. The white color denotes intermolecular distances that are close to van der Waals contacts with d_{norm} equal to zero. In turn, contacts longer than the sum of van der Waals radii with positive d_{norm} values are colored with blue.

Table S9. Results of the Hirshfeld surface analysis.

X-ray structure	Contributions of different intermolecular contacts to the molecular Hirshfeld surface*
**8**	H–H 52.7%, H–C 6.7%, H–N 5.9%, H–O 4.6%, H–M 0.5%, C–H 7.8%, C–C 0.1%, C–N 1.5%, C–O 1.1%, C–M 0.2%, N–H 7.7%, N–C 1.5%, N–N 0.7%, N–M 0.0%, O–H 5.6%, O–C 1.0%, O–O 0.7%, M–H 0.8%, M–C 0.2%, M–N 0.0%, M–M 0.6%
**9**	H–H 57.3%, H–C 5.0%, H–N 5.1%, H–O 4.5%, H–M 0.3%, C–H 5.8%, C–C 0.5%, C–N 1.8%, C–O 1.6%, C–M 0.0%, N–H 6.8%, N–C 1.1%, N–N 0.4%, N–M 0.1%, O–H 5.3%, O–C 1.4%, O–O 0.8%, M–H 0.8%, M–C 0.0%, M–N 0.1%, M–M 0.6%

*The contributions of all other intermolecular contacts do not exceed 0%.

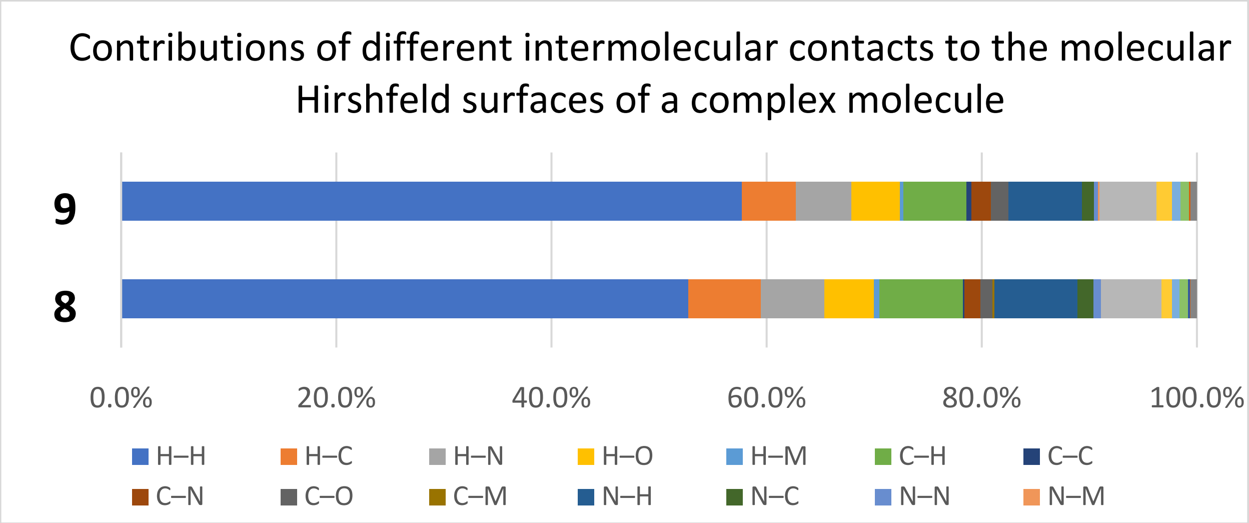

Figure S19. Contributions of various intermolecular contacts to the molecular Hirshfeld surfaces of **8** and **9** complex molecules.

8

9

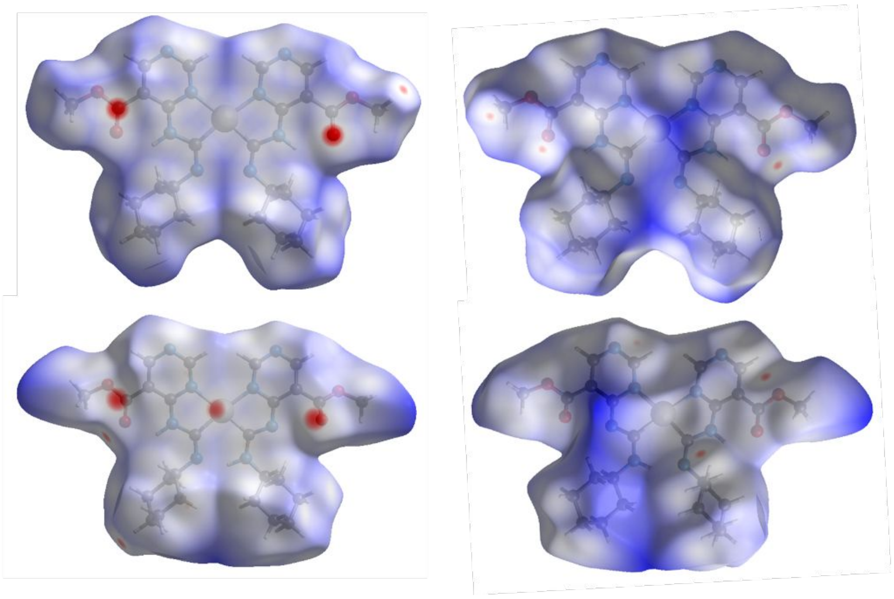

Figure S20. Hirshfeld surfaces for **8** and **9**.

S7. Details of the theoretical calculations

The single point calculations based on the experimental geometries of **8–9** and dimers of **8–9** have been carried out at the PBE0[28]-D3/def2-TZVP[29] level of theory using Gaussian-09[30] program package. The QTAIM analysis,[31] ELF,[32] NCI plots,[33] MBO[34], and WI[35] calculations have been performed by using the Multiwfn program (version 3.8).[36] The Cartesian atomic coordinates for all model structures are presented in **Table S10**.

Table S10. Cartesian atomic coordinates for model structures.

Atom	X	Y	Z
**8** (single molecule based on the experimental X-ray geometry)			
Pd	2.789100000	1.673400000	-0.125300000
Pd	-1.925200000	1.071600000	-0.058500000
S	-4.072500000	5.196900000	-1.435100000
S	4.703600000	2.960400000	-0.668500000
N	0.510700000	-0.372300000	0.220100000
N	-0.032600000	1.814900000	0.207400000
N	1.694800000	3.373500000	0.159400000
N	-1.249300000	-1.736400000	0.771600000
N	4.582400000	-0.804800000	-0.528200000
N	-2.926800000	2.841500000	-0.488500000
N	-4.671600000	-0.217700000	-0.686300000
C	0.940500000	0.897000000	0.151000000
C	-0.928200000	-0.589200000	0.373900000
N	-0.037300000	5.394900000	0.933300000
N	7.084900000	1.746400000	0.261400000
C	1.382100000	-1.506300000	0.459800000
C	-3.114400000	-3.154200000	0.240400000
C	1.845500000	-1.728400000	1.755900000
C	-2.568400000	-2.145300000	1.049600000
C	1.648100000	-2.370100000	-0.602800000
C	-5.925100000	-0.841900000	-0.794800000
C	-6.101800000	-1.803500000	-1.790400000
C	2.137000000	4.626900000	0.351900000
H	3.039600000	4.819100000	0.236900000
C	-4.384400000	-3.635800000	0.536600000
H	-4.769100000	-4.281000000	-0.011800000
C	0.384200000	3.119500000	0.338000000
C	-3.626800000	0.172900000	-0.425800000
C	5.543100000	-2.643600000	-1.753400000
C	-4.505600000	-2.216500000	2.462300000
H	-4.963500000	-1.928800000	3.218800000
C	5.483900000	-1.879000000	-0.582700000
C	4.738100000	-2.290000000	-2.975700000
H	3.802200000	-2.331600000	-2.765600000
H	4.935900000	-2.911400000	-3.680100000
H	4.962800000	-1.401100000	-3.260600000
C	2.460400000	-3.478400000	-0.351300000
H	2.668700000	-4.065100000	-1.042200000
C	-6.890700000	-0.514200000	0.170400000
C	-3.247800000	-1.679000000	2.184400000
C	-5.089300000	-3.166600000	1.642000000

H	-5.942200000	-3.487700000	1.826900000
C	6.387400000	-3.750100000	-1.738200000
H	6.449300000	-4.292100000	-2.491300000
C	3.831700000	0.049600000	-0.369600000
C	6.253100000	-2.129600000	0.560800000
C	-2.293200000	-3.698300000	-0.898200000
H	-2.141100000	-3.003900000	-1.543400000
H	-2.763900000	-4.424100000	-1.314100000
H	-1.450600000	-4.013600000	-0.562900000
C	1.266100000	5.632700000	0.718900000
H	1.590900000	6.498300000	0.820300000
C	-0.462900000	4.163100000	0.742400000
H	-1.363400000	3.976800000	0.881500000
C	2.649500000	-2.853500000	1.964000000
H	2.980900000	-3.028100000	2.815200000
C	1.500500000	-0.819400000	2.895900000
H	2.153100000	-0.117700000	2.954400000
H	1.495400000	-1.320700000	3.714700000
H	0.632100000	-0.437400000	2.749200000
C	1.094400000	-2.106800000	-1.977700000
H	0.143000000	-1.992100000	-1.923000000
H	1.294600000	-2.850400000	-2.551000000
H	1.492900000	-1.310300000	-2.336000000
C	-8.069600000	-1.257900000	0.138300000
H	-8.733400000	-1.094200000	0.768700000
C	-7.301300000	-2.502800000	-1.774900000
H	-7.459000000	-3.158700000	-2.415100000
C	2.959400000	-3.709800000	0.925200000
H	3.505600000	-4.446200000	1.081100000
C	7.087300000	-3.241100000	0.512700000
H	7.620900000	-3.443600000	1.247000000
C	-3.418000000	3.814000000	-0.885700000
C	-8.263800000	-2.235900000	-0.819600000
H	-9.056100000	-2.722900000	-0.821000000
C	-5.028700000	-2.094600000	-2.801100000
H	-4.782200000	-1.282100000	-3.249100000
H	-5.357600000	-2.727300000	-3.443800000
H	-4.260800000	-2.460300000	-2.356000000
C	6.172700000	-1.257200000	1.776200000
H	6.266700000	-0.337700000	1.517000000
H	6.876700000	-1.491600000	2.385400000
H	5.323200000	-1.383600000	2.205100000
C	-2.622300000	-0.666400000	3.105900000
H	-1.759400000	-0.979600000	3.386900000
H	-3.184000000	-0.543500000	3.874700000
H	-2.524600000	0.169300000	2.643900000
C	6.062800000	2.190700000	-0.096400000
C	7.137700000	-4.053100000	-0.610700000
H	7.680200000	-4.808600000	-0.608300000
C	-6.647100000	0.552700000	1.190100000
H	-5.941900000	0.276500000	1.780000000
H	-7.448900000	0.700100000	1.696900000
H	-6.393900000	1.366300000	0.747800000
**9** (single molecule based on the experimental X-ray geometry)			
Pt	1.981400000	0.941900000	-0.074200000

Pt	-2.742500000	1.438400000	-0.119100000
S	4.043600000	5.091400000	-1.438000000
S	-4.731200000	2.623600000	-0.546900000
N	-0.444900000	-0.534300000	0.219000000
N	1.310900000	-1.890300000	0.784700000
N	0.089100000	1.668200000	0.170100000
N	2.923600000	2.730700000	-0.546500000
N	-1.693600000	3.162900000	0.176100000
N	4.734100000	-0.288100000	-0.633700000
N	-4.496200000	-1.043900000	-0.536900000
C	1.001500000	-0.720400000	0.371900000
N	-0.025400000	5.250400000	0.936900000
C	4.478900000	-3.727200000	0.549500000
H	4.873000000	-4.368900000	0.003400000
C	-0.894300000	0.725000000	0.116500000
C	4.576600000	-2.315400000	2.473400000
H	5.028400000	-2.024000000	3.231900000
C	3.196600000	-3.262400000	0.252500000
C	3.677200000	0.112600000	-0.394800000
C	5.985900000	-0.914800000	-0.743100000
C	-1.304700000	-1.671300000	0.446700000
C	5.101800000	-2.153300000	-2.764600000
H	4.326500000	-2.508300000	-2.323300000
H	5.431100000	-2.794100000	-3.398400000
H	4.865300000	-1.342700000	-3.222600000
C	-2.860200000	-3.894300000	0.892600000
H	-3.390500000	-4.643500000	1.042400000
C	0.444200000	4.032200000	0.702700000
H	1.356300000	3.883400000	0.803100000
C	6.173100000	-1.858800000	-1.741100000
C	-0.364500000	2.966800000	0.313500000
C	-5.393800000	-2.109700000	-0.590200000
C	-1.777800000	-1.895200000	1.740400000
C	-2.349800000	-3.652000000	-0.370500000
H	-2.546200000	-4.238600000	-1.064100000
C	-1.548900000	-2.543600000	-0.619600000
C	-3.751200000	-0.175800000	-0.366500000
C	3.309000000	-1.801400000	2.199400000
C	2.643700000	-2.268800000	1.063200000
C	-6.163600000	-2.349700000	0.548200000
C	5.174000000	-3.241600000	1.655500000
H	6.034900000	-3.541700000	1.837100000
C	8.345200000	-2.287900000	-0.776700000
H	9.141000000	-2.769500000	-0.782900000
C	-0.988100000	-2.259700000	-1.995400000
H	-1.428600000	-1.493300000	-2.368600000
H	-1.132900000	-3.020600000	-2.562600000
H	-0.046500000	-2.086400000	-1.926500000
C	-2.583000000	-3.024100000	1.933900000
H	-2.937300000	-3.193800000	2.775900000
C	2.394900000	-3.811400000	-0.886100000
H	2.224200000	-3.112500000	-1.522400000
H	1.561600000	-4.153100000	-0.554900000
H	2.885300000	-4.518200000	-1.311300000
C	-7.064500000	-4.263900000	-0.612800000

H	-7.617800000	-5.011800000	-0.613900000
C	-1.460000000	-0.976400000	2.869200000
H	-0.551500000	-0.674300000	2.788700000
H	-1.569100000	-1.441900000	3.701300000
H	-2.051200000	-0.221200000	2.843800000
C	3.396400000	3.695900000	-0.913000000
C	-5.459400000	-2.874500000	-1.759300000
C	8.133600000	-1.311000000	0.194700000
H	8.793400000	-1.140700000	0.827700000
C	7.380200000	-2.546300000	-1.732700000
H	7.542600000	-3.192900000	-2.380900000
C	6.940500000	-0.585500000	0.224600000
C	2.672900000	-0.808300000	3.139900000
H	3.216600000	-0.719800000	3.926300000
H	1.799300000	-1.119100000	3.388200000
H	2.598800000	0.042800000	2.703700000
C	-6.319000000	-3.973300000	-1.734700000
H	-6.388200000	-4.518200000	-2.485500000
C	-2.184900000	4.396100000	0.388400000
H	-3.095500000	4.552800000	0.288000000
C	-7.007100000	-3.466800000	0.511700000
H	-7.532200000	-3.672900000	1.252000000
C	-1.358400000	5.415800000	0.750200000
H	-1.723500000	6.262200000	0.875800000
C	-4.643500000	-2.525900000	-2.976200000
H	-4.856800000	-1.633400000	-3.260000000
H	-4.845600000	-3.142500000	-3.684400000
H	-3.709800000	-2.579100000	-2.762400000
C	-6.095400000	-1.460300000	1.751900000
H	-5.264200000	-1.603800000	2.209600000
H	-6.825100000	-1.663400000	2.342400000
H	-6.154400000	-0.542300000	1.475900000
C	6.702700000	0.471300000	1.254200000
H	6.446200000	1.288800000	0.820900000
H	7.507800000	0.615400000	1.757400000
H	6.001700000	0.189500000	1.846400000
N	-7.060200000	1.565400000	0.529900000
C	-5.984800000	1.982200000	0.069300000
**7** (dimer molecular associate based on the experimental X-ray geometry)			
Pt	3.092445000	7.164821000	4.536213000
Cl	1.668025000	6.207203000	2.893880000
N	3.838878000	8.610325000	3.305505000
O	6.957873000	11.056937000	4.848604000
O	6.818399000	12.304416000	2.988947000
N	5.029557000	9.169147000	5.243558000
N	2.259300000	5.193640000	6.720169000
N	4.151926000	9.697145000	1.230509000
N	4.583441000	7.781488000	6.989095000
H	4.156814000	7.088059000	7.322047000
C	6.463900000	11.350809000	3.645880000
C	5.066507000	8.271697000	9.323093000
H	4.133434000	8.581255000	9.440220000
H	5.101139000	7.306268000	9.540417000
C	5.986577000	9.044167000	10.265822000
H	5.719989000	8.870060000	11.202944000

H	5.892391000	10.014958000	10.095575000
C	4.777556000	9.399473000	3.949538000
C	5.506416000	8.491256000	7.871291000
H	5.441778000	9.468550000	7.669378000
C	4.338230000	8.099849000	5.724846000
C	1.850847000	4.290490000	7.780987000
H	2.338759000	3.423926000	7.674399000
C	5.421991000	10.389602000	3.163600000
C	2.544365000	5.906787000	5.881636000
C	-0.463399000	5.277875000	7.940755000
H	-1.429975000	5.072418000	7.889093000
H	-0.258537000	5.969931000	7.262466000
C	2.203595000	4.895757000	9.155387000
H	2.060218000	4.214299000	9.859695000
H	3.160415000	5.149095000	9.166124000
C	5.079650000	10.473415000	1.828894000
H	5.527760000	11.119343000	1.296659000
C	7.867812000	8.818274000	8.601657000
H	7.844177000	9.782478000	8.375879000
H	8.796458000	8.499110000	8.480671000
C	6.944945000	8.056148000	7.657936000
H	7.209530000	8.232282000	6.720258000
H	7.026777000	7.084703000	7.825018000
C	3.567840000	8.811307000	2.010494000
H	2.894702000	8.267095000	1.618963000
C	0.338284000	4.030185000	7.651982000
H	0.136959000	3.717102000	6.734429000
H	0.072826000	3.316596000	8.283859000
C	7.452860000	8.630381000	10.067353000
H	8.034831000	9.181856000	10.649166000
H	7.568776000	7.682623000	10.325452000
C	1.356070000	6.116002000	9.457060000
H	1.595132000	6.844876000	8.830433000
H	1.547289000	6.429782000	10.376598000
C	-0.127307000	5.809885000	9.329047000
H	-0.649668000	6.632579000	9.500813000
H	-0.383566000	5.136745000	10.008437000
C	7.956295000	11.982887000	5.354700000
H	8.667626000	12.091232000	4.690047000
H	8.336796000	11.628721000	6.185939000
H	7.537045000	12.850274000	5.530723000
Pt	4.584029000	13.946679000	5.387249000
Cl	6.008449000	14.904297000	7.029583000
N	3.837597000	12.501175000	6.617957000
O	0.718602000	10.054563000	5.074859000
O	0.858076000	8.807084000	6.934516000
N	2.646917000	11.942353000	4.679905000
N	5.417174000	15.917860000	3.203294000
N	3.524549000	11.414355000	8.692953000
N	3.093034000	13.330012000	2.934368000
H	3.519661000	14.023441000	2.601416000
C	1.212575000	9.760691000	6.277583000
C	2.609968000	12.839803000	0.600369000
H	3.543041000	12.530245000	0.483243000
H	2.575335000	13.805232000	0.383046000

C	1.689898000	12.067333000	-0.342359000
H	1.956486000	12.241440000	-1.279482000
H	1.784084000	11.096542000	-0.172113000
C	2.898918000	11.712027000	5.973925000
C	2.170059000	12.620244000	2.052172000
H	2.234696000	11.642950000	2.254085000
C	3.338244000	13.011651000	4.198617000
C	5.825627000	16.821010000	2.142476000
H	5.337716000	17.687574000	2.249064000
C	2.254484000	10.721898000	6.759863000
C	5.132110000	15.204713000	4.041826000
C	8.139874000	15.833625000	1.982708000
H	9.106449000	16.039082000	2.034369000
H	7.935011000	15.141569000	2.660996000
C	5.472880000	16.215743000	0.768076000
H	5.616257000	16.897201000	0.063768000
H	4.516060000	15.962405000	0.757339000
C	2.596824000	10.638085000	8.094569000
H	2.148714000	9.992157000	8.626804000
C	-0.191338000	12.293226000	1.321805000
H	-0.167703000	11.329022000	1.547584000
H	-1.119984000	12.612390000	1.442792000
C	0.731530000	13.055352000	2.265527000
H	0.466945000	12.879218000	3.203204000
H	0.649698000	14.026797000	2.098445000
C	4.108634000	12.300193000	7.912969000
H	4.781773000	12.844405000	8.304499000
C	7.338190000	17.081315000	2.271481000
H	7.539515000	17.394398000	3.189034000
H	7.603649000	17.794904000	1.639604000
C	0.223615000	12.481119000	-0.143890000
H	-0.358356000	11.929644000	-0.725703000
H	0.107698000	13.428877000	-0.401990000
C	6.320405000	14.995498000	0.466403000
H	6.081343000	14.266624000	1.093030000
H	6.129185000	14.681718000	-0.453135000
C	7.803782000	15.301615000	0.594415000
H	8.326142000	14.478921000	0.422650000
H	8.060041000	15.974755000	-0.084975000
C	-0.279820000	9.128613000	4.568762000
H	-0.991151000	9.020268000	5.233416000
H	-0.660321000	9.482779000	3.737523000
H	0.139429000	8.261226000	4.392740000
**8** (dimer molecular associate based on the experimental X-ray geometry)			
Pd	-1.442256000	3.720750000	6.734597000
O	-0.844322000	3.144056000	0.214842000
N	-1.679383000	4.397431000	4.711314000
N	-2.265762000	5.242616000	7.979946000
N	-0.444890000	1.872977000	8.794661000
N	0.016486000	1.196994000	6.241670000
O	-0.136532000	1.703105000	1.772612000
N	-0.629253000	2.372086000	4.364012000
H	-0.318805000	1.737705000	3.838702000
N	-1.592230000	3.713487000	9.590782000
H	-1.531629000	3.429010000	10.421498000

N	-3.250278000	7.336755000	8.555661000
O	-2.755678000	6.489976000	12.487954000
N	-2.274396000	5.829926000	2.896222000
C	-1.170747000	3.481335000	3.842176000
C	-1.219299000	3.721208000	2.446995000
C	-2.657007000	5.777930000	10.286065000
C	-1.090701000	2.971247000	8.544804000
C	-2.174561000	4.879979000	9.298039000
C	0.623800000	0.178471000	5.377146000
H	0.006323000	0.007998000	4.609267000
C	-0.237289000	1.325867000	10.143562000
H	-1.068644000	1.465869000	10.680828000
C	-0.565993000	2.235521000	5.749013000
C	-1.774910000	4.917979000	2.047984000
H	-1.805700000	5.108459000	1.118208000
C	-2.600129000	5.438313000	11.726033000
C	-3.166649000	6.980362000	9.858554000
H	-3.477219000	7.595610000	10.512294000
C	0.930813000	1.998302000	10.856081000
H	0.750875000	2.966938000	10.951396000
H	1.756327000	1.888918000	10.319914000
C	-0.688971000	2.741718000	1.480683000
C	0.021892000	-0.174002000	10.013272000
H	0.822618000	-0.322962000	9.450436000
H	-0.750478000	-0.604607000	9.567398000
C	-2.803722000	6.434242000	7.697924000
H	-2.874298000	6.657767000	6.777824000
C	-2.201130000	5.505776000	4.180114000
H	-2.562721000	6.134731000	4.793355000
C	1.971866000	0.626610000	4.820703000
H	1.866464000	1.490691000	4.350562000
H	2.611789000	0.756158000	5.565306000
C	0.238047000	-0.813257000	11.390810000
H	-0.594753000	-0.741998000	11.920408000
H	0.449064000	-1.773472000	11.277879000
C	1.129023000	1.367303000	12.248547000
H	1.903034000	1.791841000	12.696390000
H	0.325480000	1.532135000	12.802792000
C	0.800933000	-1.122763000	6.146667000
H	-0.068179000	-1.410180000	6.524601000
H	1.433564000	-0.985557000	6.896183000
C	1.339732000	-2.204274000	5.204784000
H	1.490362000	-3.039789000	5.714731000
H	0.668959000	-2.392023000	4.501426000
C	2.514009000	-0.417610000	3.857105000
H	3.399227000	-0.129348000	3.519425000
H	1.902976000	-0.504127000	3.083173000
C	1.368055000	-0.133939000	12.137259000
H	2.221400000	-0.296405000	11.660889000
H	1.444766000	-0.523520000	13.043597000
C	2.648658000	-1.765734000	4.553338000
H	3.353773000	-1.705407000	5.245771000
H	2.928795000	-2.447179000	3.892188000
C	-0.426326000	2.215498000	-0.812951000
H	-0.841579000	2.462658000	-1.665341000

H	-0.703747000	1.308060000	-0.565822000
H	0.548875000	2.245042000	-0.903542000
C	-2.686170000	6.312642000	13.913807000
H	-2.488537000	5.373980000	14.117357000
H	-3.545382000	6.559120000	14.316346000
H	-1.978355000	6.881980000	14.280788000
O	-2.413467000	4.348730000	12.172545000
Pd	-4.246860000	1.913781000	6.837200000
O	-4.844794000	2.490475000	13.356956000
N	-4.009732000	1.237099000	8.860484000
N	-3.423354000	0.391915000	5.591852000
N	-5.244226000	3.761553000	4.777137000
N	-5.705602000	4.437536000	7.330128000
O	-5.552584000	3.931426000	11.799185000
N	-5.059863000	3.262444000	9.207786000
H	-5.370310000	3.896826000	9.733096000
N	-4.096886000	1.921043000	3.981015000
H	-4.157487000	2.205520000	3.150299000
N	-2.438838000	-1.702225000	5.016136000
O	-2.933438000	-0.855445000	1.083844000
N	-3.414720000	-0.195396000	10.675576000
C	-4.518369000	2.153195000	9.729622000
C	-4.469817000	1.913323000	11.124803000
C	-3.032108000	-0.143399000	3.285732000
C	-4.598415000	2.663283000	5.026994000
C	-3.514555000	0.754552000	4.273759000
C	-6.312916000	5.456060000	8.194651000
H	-5.695439000	5.626533000	8.962530000
C	-5.451827000	4.308663000	3.428236000
H	-4.620472000	4.168662000	2.890969000
C	-5.123123000	3.399010000	7.822784000
C	-3.914206000	0.716551000	11.523813000
H	-3.883416000	0.526072000	12.453590000
C	-3.088987000	0.196217000	1.845764000
C	-2.522466000	-1.345832000	3.713244000
H	-2.211897000	-1.961079000	3.059504000
C	-6.619928000	3.636228000	2.715717000
H	-6.439991000	2.667593000	2.620402000
H	-7.445443000	3.745613000	3.251884000
C	-5.000145000	2.892812000	12.091115000
C	-5.711008000	5.808533000	3.558525000
H	-6.511734000	5.957493000	4.121361000
H	-4.938637000	6.239138000	4.004400000
C	-2.885394000	-0.799711000	5.873874000
H	-2.814818000	-1.023237000	6.793974000
C	-3.487986000	0.128754000	9.391684000
H	-3.126395000	-0.500201000	8.778442000
C	-7.660982000	5.007920000	8.751095000
H	-7.555580000	4.143840000	9.221236000
H	-8.300905000	4.878372000	8.006492000
C	-5.927163000	6.447788000	2.180988000
H	-5.094363000	6.376529000	1.651389000
H	-6.138180000	7.408003000	2.293919000
C	-6.818138000	4.267228000	1.323250000
H	-7.592150000	3.842689000	0.875408000

H	-6.014596000	4.102395000	0.769005000
C	-6.490049000	6.757293000	7.425131000
H	-5.620936000	7.044711000	7.047197000
H	-7.122680000	6.620088000	6.675614000
C	-7.028848000	7.838804000	8.367013000
H	-7.179478000	8.674319000	7.857067000
H	-6.358075000	8.026553000	9.070372000
C	-8.203125000	6.052140000	9.714693000
H	-9.088343000	5.763879000	10.052373000
H	-7.592092000	6.138657000	10.488625000
C	-7.057171000	5.768469000	1.434539000
H	-7.910516000	5.930936000	1.910909000
H	-7.133881000	6.158051000	0.528201000
C	-8.337774000	7.400265000	9.018460000
H	-9.042889000	7.339938000	8.326026000
H	-8.617911000	8.081709000	9.679610000
C	-5.262790000	3.419032000	14.384748000
H	-4.847537000	3.171873000	15.237139000
H	-4.985369000	4.326471000	14.137619000
H	-6.237991000	3.389489000	14.475340000
C	-3.002946000	-0.678111000	-0.342009000
H	-3.200579000	0.260550000	-0.545559000
H	-2.143734000	-0.924590000	-0.744549000
H	-3.710761000	-1.247450000	-0.708991000
O	-3.275649000	1.285801000	1.399252000
**9** (dimer molecular associate based on the experimental X-ray geometry)			
Pt	3.379605000	2.272715000	7.630332000
O	2.923799000	-2.870961000	3.608819000
N	4.020000000	1.025088000	6.026327000
N	2.009427000	0.044600000	6.514738000
H	1.398596000	-0.579152000	6.401900000
N	4.987346000	3.591347000	8.017444000
N	3.475805000	4.368273000	9.628685000
N	1.569893000	3.095622000	9.766939000
H	1.084319000	2.426189000	9.466967000
O	6.425159000	6.845111000	11.073246000
O	1.482287000	-2.278343000	5.225228000
N	0.698411000	1.118830000	8.122103000
N	7.209301000	4.447134000	7.866269000
N	5.423752000	0.240700000	4.275539000
C	4.651026000	4.476169000	9.020109000
O	4.310683000	7.090739000	10.531859000
C	2.485365000	-2.097397000	4.599855000
C	2.715446000	3.326606000	9.166115000
C	3.127126000	0.029426000	5.780829000
C	1.812421000	1.061072000	7.470888000
C	3.402234000	-0.943715000	4.798837000
C	5.635239000	5.441273000	9.390940000
C	-0.403712000	0.182052000	7.884358000
H	-0.446637000	-0.021499000	6.905558000
C	1.037666000	3.856241000	10.891061000
H	1.276910000	4.819059000	10.764345000
C	4.564681000	-0.773388000	4.071388000
H	4.767114000	-1.405104000	3.391075000
C	5.349523000	6.502010000	10.392313000

C	6.223000000	3.627868000	7.521280000
H	6.422147000	2.994207000	6.841690000
C	6.885923000	5.358171000	8.801745000
H	7.551869000	5.978453000	9.072426000
C	5.106566000	1.075158000	5.236857000
H	5.709723000	1.791799000	5.392593000
C	-1.708830000	0.849405000	8.304289000
H	-1.666539000	1.077987000	9.266744000
H	-1.828789000	1.688962000	7.794441000
C	-0.484454000	3.733754000	10.905274000
H	-0.849754000	4.055978000	10.043925000
H	-0.737600000	2.782016000	11.009236000
C	2.093377000	-3.996614000	3.258661000
H	2.575468000	-4.575996000	2.632655000
H	1.268647000	-3.675171000	2.837077000
H	1.870658000	-4.502569000	4.067551000
C	-0.217596000	-1.120347000	8.645401000
H	-0.117321000	-0.923606000	9.610686000
H	0.610070000	-1.565895000	8.336436000
C	1.611335000	3.386783000	12.220626000
H	2.596765000	3.481628000	12.209643000
H	1.394628000	2.430976000	12.357639000
C	-2.896538000	-0.070866000	8.053622000
H	-2.976341000	-0.250655000	7.083208000
H	-3.728249000	0.373033000	8.353827000
C	-1.082526000	4.542070000	12.042317000
H	-0.913273000	5.504463000	11.883221000
H	-2.063050000	4.404363000	12.060690000
C	-2.722224000	-1.389364000	8.803037000
H	-2.748057000	-1.218615000	9.778284000
H	-3.470408000	-1.996738000	8.578639000
C	-1.401894000	-2.056978000	8.442543000
H	-1.430862000	-2.345467000	7.495347000
H	-1.280159000	-2.863561000	9.002782000
C	6.270071000	7.904693000	12.042317000
H	6.245613000	8.768368000	11.580083000
H	7.026341000	7.889061000	12.665597000
H	5.435263000	7.774523000	12.537525000
C	-0.500319000	4.148556000	13.377050000
H	-0.784073000	3.226288000	13.598709000
H	-0.851934000	4.752492000	14.078463000
C	1.026465000	4.218719000	13.370589000
H	1.375329000	3.879243000	14.232843000
H	1.312386000	5.161329000	13.274096000
Pt	2.180002000	4.150712000	5.290608000
O	2.635808000	9.294388000	9.312122000
N	1.539607000	5.398338000	6.894614000
N	3.550180000	6.378826000	6.406202000
H	4.161012000	7.002578000	6.519041000
N	0.572261000	2.832079000	4.903497000
N	2.083802000	2.055154000	3.292256000
N	3.989714000	3.327804000	3.154002000
H	4.475288000	3.997237000	3.453974000
O	-0.865552000	-0.421684000	1.847695000
O	4.077320000	8.701769000	7.695712000

N	4.861196000	5.304597000	4.798837000
N	-1.649694000	1.976292000	5.054672000
N	0.135856000	6.182727000	8.645401000
C	0.908581000	1.947258000	3.900832000
O	1.248924000	-0.667312000	2.389082000
C	3.074242000	8.520824000	8.321086000
C	2.844161000	3.096820000	3.754825000
C	2.432482000	6.394001000	7.140112000
C	3.747187000	5.362354000	5.450053000
C	2.157374000	7.367142000	8.122103000
C	-0.075632000	0.982153000	3.530001000
C	5.963320000	6.241374000	5.036583000
H	6.006244000	6.444925000	6.015383000
C	4.521941000	2.567186000	2.029880000
H	4.282697000	1.604368000	2.156595000
C	0.994926000	7.196814000	8.849552000
H	0.792493000	7.828531000	9.529866000
C	0.210085000	-0.078584000	2.528628000
C	-0.663393000	2.795558000	5.399661000
H	-0.862540000	3.429220000	6.079251000
C	-1.326316000	1.065256000	4.119196000
H	-1.992262000	0.444974000	3.848515000
C	0.453042000	5.348269000	7.684083000
H	-0.150116000	4.631628000	7.528347000
C	7.268437000	5.574021000	4.616652000
H	7.226146000	5.345440000	3.654197000
H	7.388397000	4.734464000	5.126500000
C	6.044061000	2.689672000	2.015667000
H	6.409361000	2.367449000	2.877015000
H	6.297207000	3.641411000	1.911705000
C	3.466230000	10.420041000	9.662279000
H	2.984139000	10.999422000	10.288286000
H	4.290960000	10.098598000	10.083864000
H	3.688949000	10.925995000	8.853390000
C	5.777203000	7.543773000	4.275539000
H	5.676928000	7.347033000	3.310255000
H	4.949537000	7.989321000	4.584505000
C	3.948272000	3.036644000	0.700315000
H	2.962843000	2.941798000	0.711298000
H	4.164979000	3.992450000	0.563301000
C	8.456145000	6.494292000	4.867318000
H	8.535948000	6.674081000	5.837733000
H	9.287856000	6.050394000	4.567113000
C	6.642133000	1.881357000	0.878624000
H	6.472880000	0.918964000	1.037720000
H	7.622657000	2.019064000	0.860250000
C	8.281831000	7.812791000	4.117904000
H	8.307664000	7.642041000	3.142657000
H	9.030015000	8.420164000	4.342302000
C	6.961502000	8.480404000	4.478398000
H	6.990469000	8.768893000	5.425593000
H	6.839767000	9.286988000	3.918159000
C	-0.710464000	-1.481267000	0.878624000
H	-0.686006000	-2.344941000	1.340858000
H	-1.466734000	-1.465635000	0.255344000

H	0.124344000	-1.351096000	0.383416000
C	6.059926000	2.274871000	-0.456109000
H	6.343680000	3.197139000	-0.677768000
H	6.411542000	1.670935000	-1.157522000
C	4.533142000	2.204708000	-0.449649000
H	4.184278000	2.544183000	-1.311902000
H	4.247221000	1.262097000	-0.353155000

Table S11. Characteristic parameters of hydrogen bonds in the **8–9**, where values of the density of all electrons – ρ(**r**), Laplacian of electron density – $\nabla^2\rho(\mathbf{r})$, energy density – H_b, potential energy density – V(**r**), and Lagrangian kinetic energy – G(**r**) (a.u.) at the bond critical points (3, –1).

Contact	*d*(H···X), Å	*d*(N···X), Å	*d*(H···X) /Σ(R_{vdW}) (Bondi)	*d*(H···X) /Σ(R_{vdW}) (Alvarez)	ρ(**r**)	$\nabla^2\rho(\mathbf{r})$	H_b	V(**r**)	G(**r**)
**8**									
N–H···O=C	2.074(3)	2.721(4)	0.76	0.77	0.022	0.098	0.003	-0.018	0.021
N–H···O=C	2.166(3)	2.783(4)	0.80	0.80	0.018	0.082	0.003	-0.014	0.017
**9**									
N–H···O=C	2.069(3)	2.709(4)	0.76	0.77	0.022	0.099	0.003	-0.018	0.022
N–H···N=C	1.915(3)	2.715(4)	0.70	0.67	0.009	0.032	0.002	-0.005	0.007

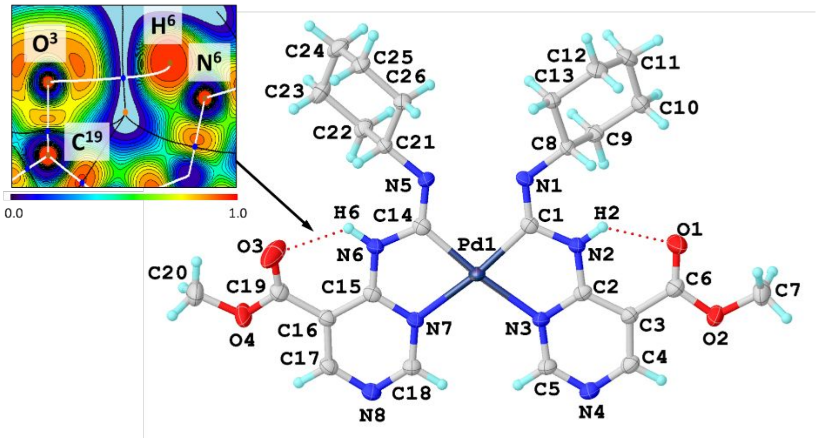

Figure S21. Visualization of electron localization function (ELF) for N–H···O=C HBs in **8**. Bond critical points (3, –1) are shown in blue, nuclear critical points (3, –3) – in pale brown, bond paths are coloured in white, colour scale for the is presented in a.u..

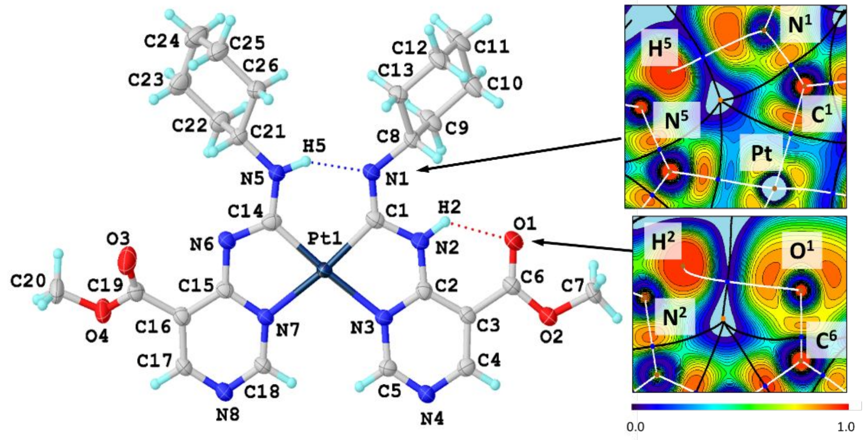

Figure S22. Visualization of electron localization function (ELF) for N–H···O=C HBs and N–H···N=C HBs in **9**. Bond critical points (3, –1) are shown in blue, nuclear critical points (3, –3) – in pale brown, bond paths are coloured in white, colour scale for the is presented in a.u..

Table S12. Characteristic parameters of noncovalent contacts in the **8–9** structures, where values of the density of all electrons – ρ(**r**), Laplacian of electron density – $\nabla^2\rho(\mathbf{r})$, energy density – H_b, potential energy density – V(**r**), and Lagrangian kinetic energy – G(**r**) (a.u.) at the bond critical points (3, –1).

Contact	*d*, Å	*d*/Σ(R_{vdW}) (Bondi)[27]	*d*/Σ(R_{vdW}) (Alvarez)[37]	ρ(**r**)	∇^2ρ(**r**)	H_b	V(**r**)	G(**r**)
**8**								
Pd···Pd	3.3379(5)	1.02	0.78	0.014	0.034	-0.003	-0.009	0.009
N6···C2	3.309(4)	1.02	0.96	0.005	0.018	0.001	-0.002	0.003
N2···C15	3.319(4)	1.02	0.97	0.005	0.019	0.001	-0.003	0.004
N3···C14	3.410(4)	1.05	0.99	0.005	0.017	0.001	-0.002	0.003
O3···C6	2.969(5)	0.92	0.91	0.007	0.031	0.002	-0.005	0.006
**9**								
Pt···Pt	3.2311(5)	0.94	0.71	0.023	0.052	-0.002	-0.016	0.014
N6···C2	3.374(6)	1.04	0.98	0.005	0.018	0.001	-0.002	0.003
N2···C15	3.415(6)	1.05	1.00	0.004	0.017	0.001	-0.002	0.003

Table S13. Calculated bond order for M···M interactions. MBO – Mayer bond order, WBI – Wiberg bond indices, ρ(**r**) – density of all electrons.

Contact	MBO	WBI	ρ(**r**)
Pd···Pd	0.157	0.178	0.014
Pt···Pt	0.435	0.235	0.023

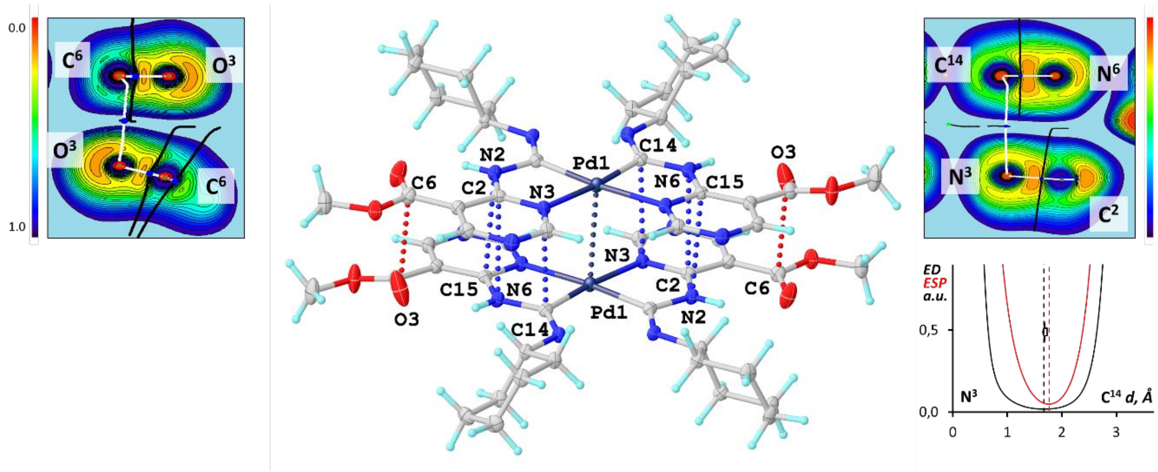

Figure S23. Visualization of electron localization function (ELF) for O3···C6 intermolecular contacts and N3···C14 in (**8**)$_2$. Bond critical points (3, –1) are shown in blue, nuclear critical points (3, –3) – in pale brown, bond paths are coloured in white, colour scale for the is presented in a.u..

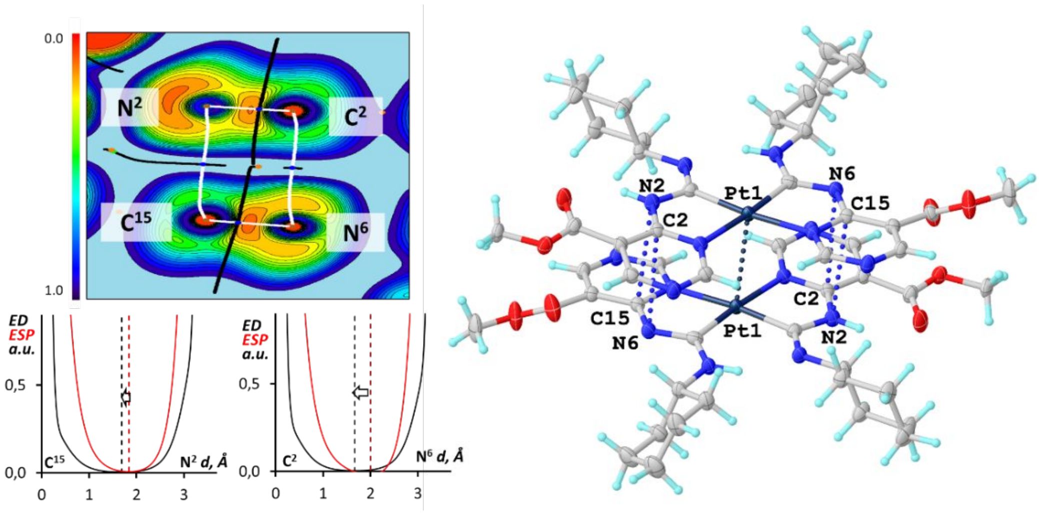

Figure S24. Visualization of electron localization function (ELF) for for the N6···C2, N2···C15 intermolecular contacts in (**9**)$_2$ Bond critical points (3, –1) are shown in blue, nuclear critical points (3, –3) – in pale brown, bond paths are coloured in white, colour scale for the is presented in a.u... The diagrams for N6···C2 and N2···C15 interactions in (**8**)$_2$ look identical.

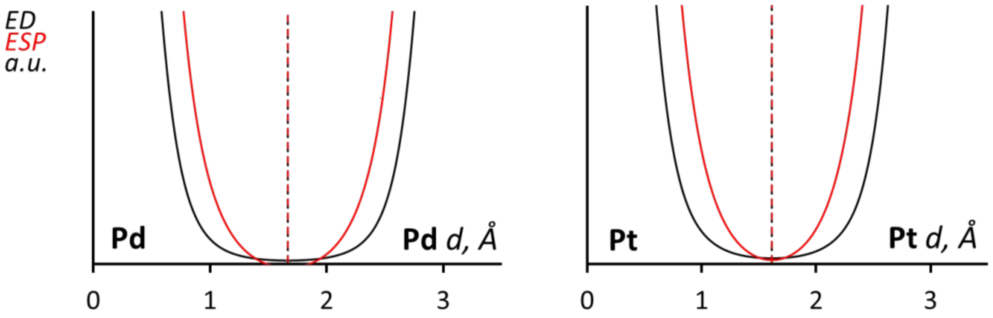

Figure S25. The ED and ESP 1D profiles along the M···M bond paths for the (**8**)$_2$ (left) and (**9**)$_2$ (right) dimeric clusters.

S8. NMR and FTIR spectra

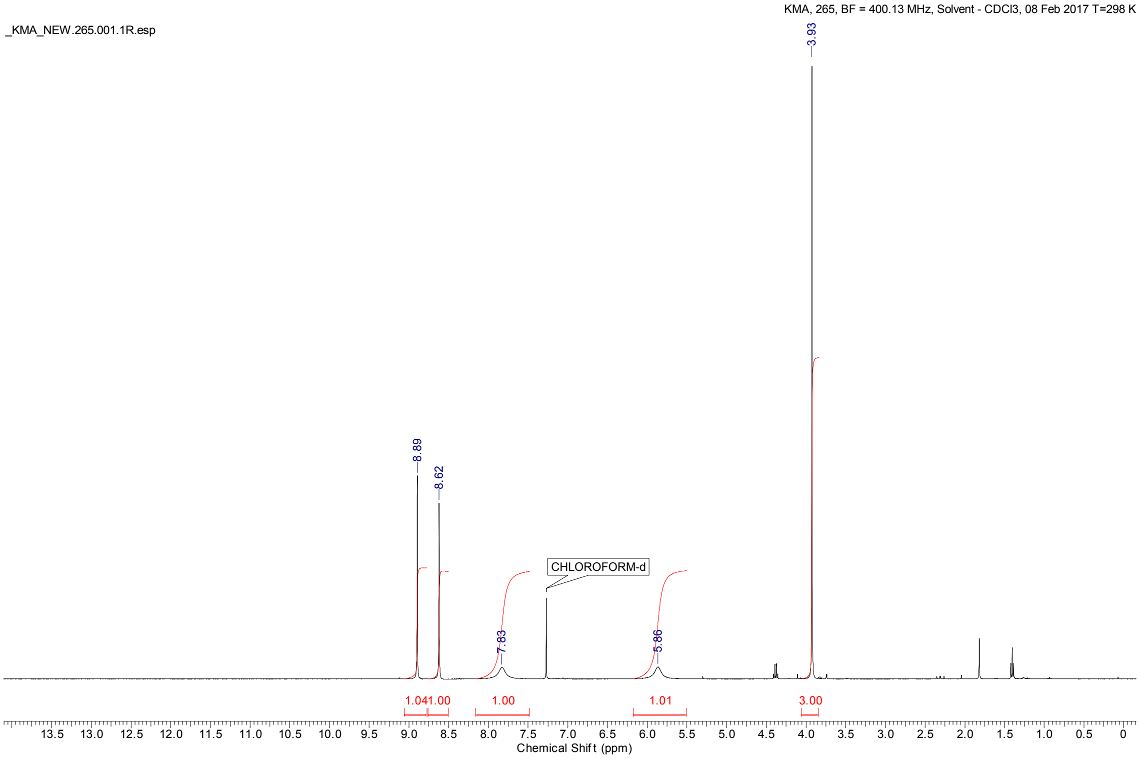

Figure S26. ^{1}H NMR spectra of **3** in $CDCl_3$ at RT.

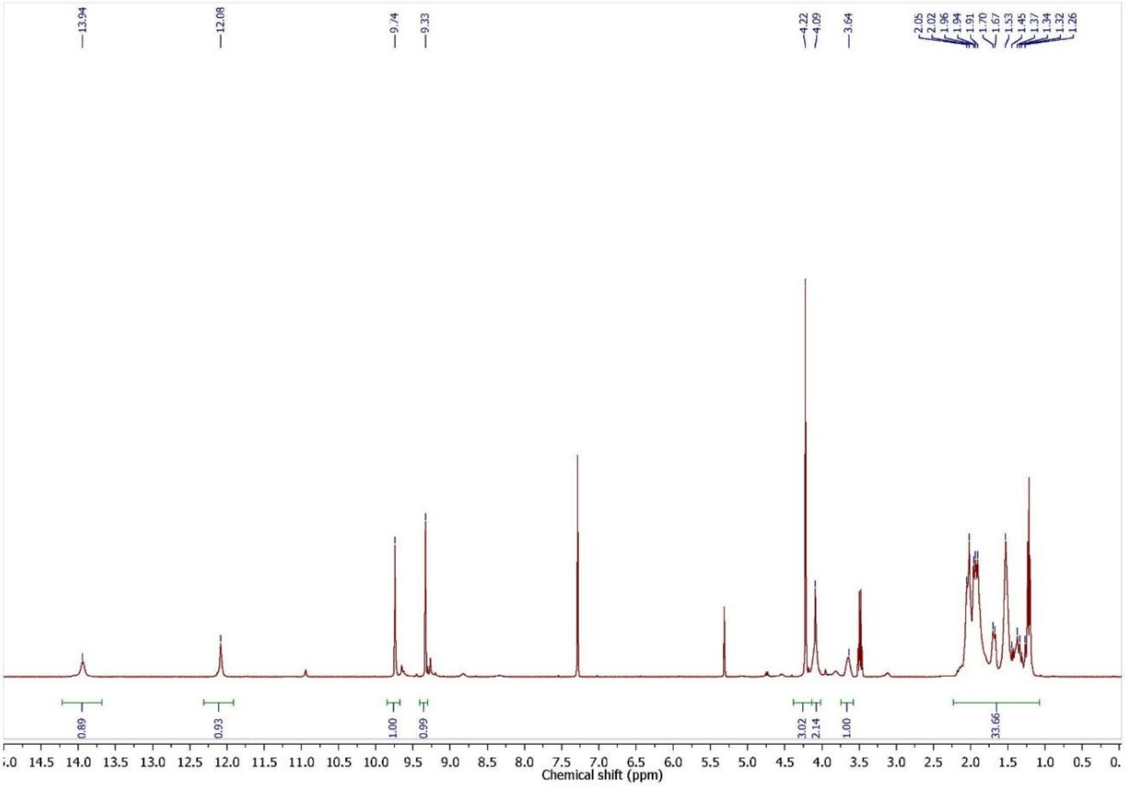

Figure S27. ^{1}H NMR spectra of **4** in $CDCl_3$ at RT.

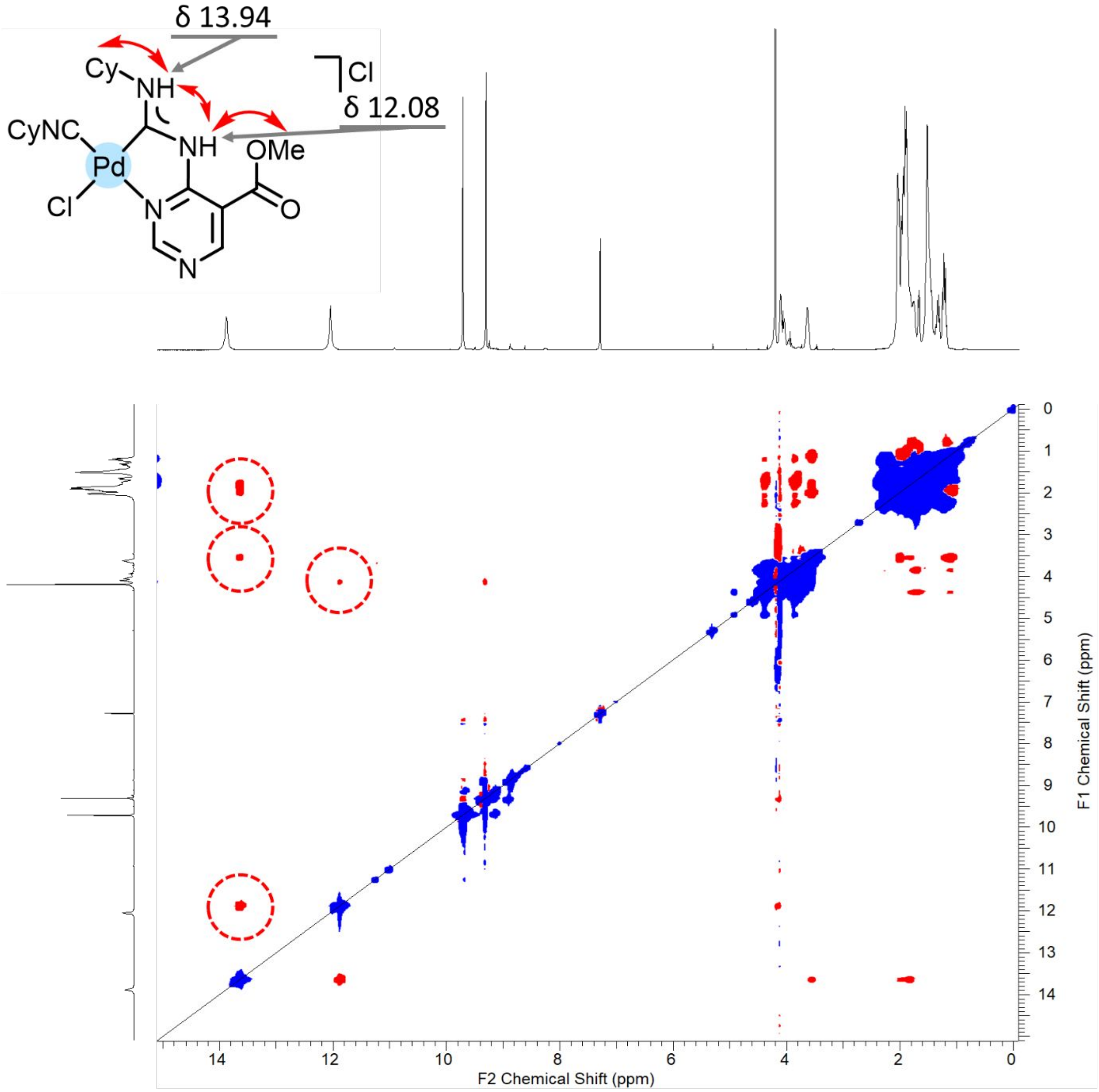

Figure S28. $^{1}H,^{1}H$-ROESY NMR spectrum of **4** in $CDCl_3$.

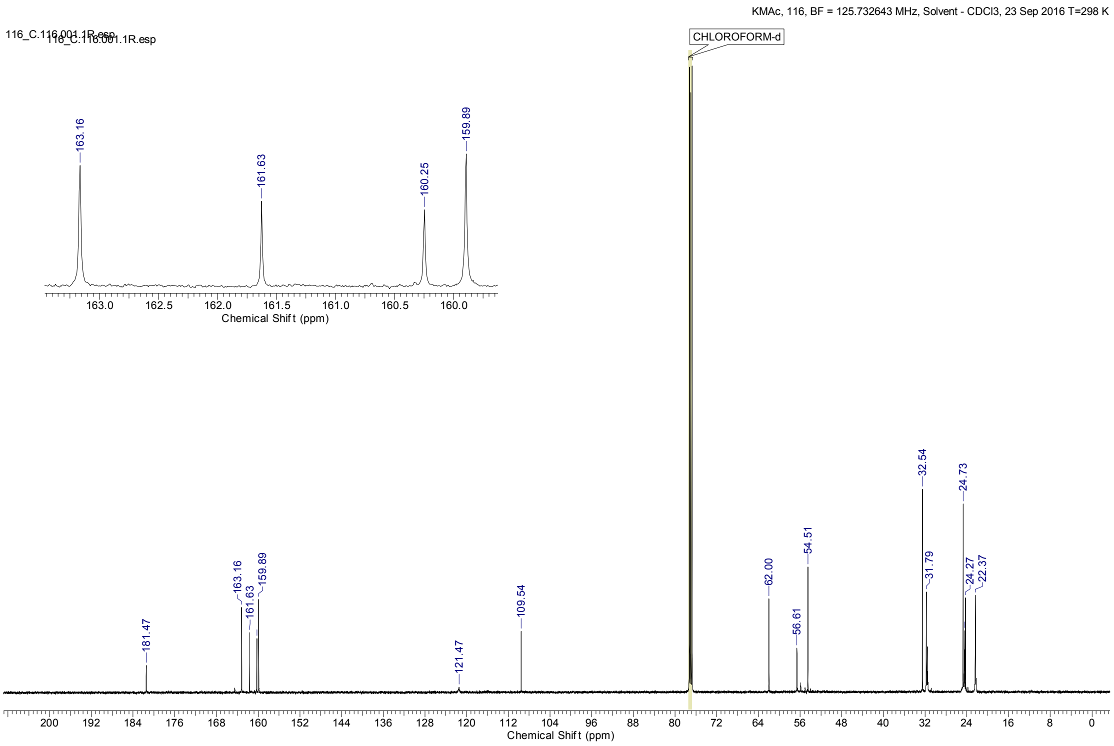

Figure S29. $^{13}C\{^1H\}$ NMR spectra of **4** in $CDCl_3$ at RT.

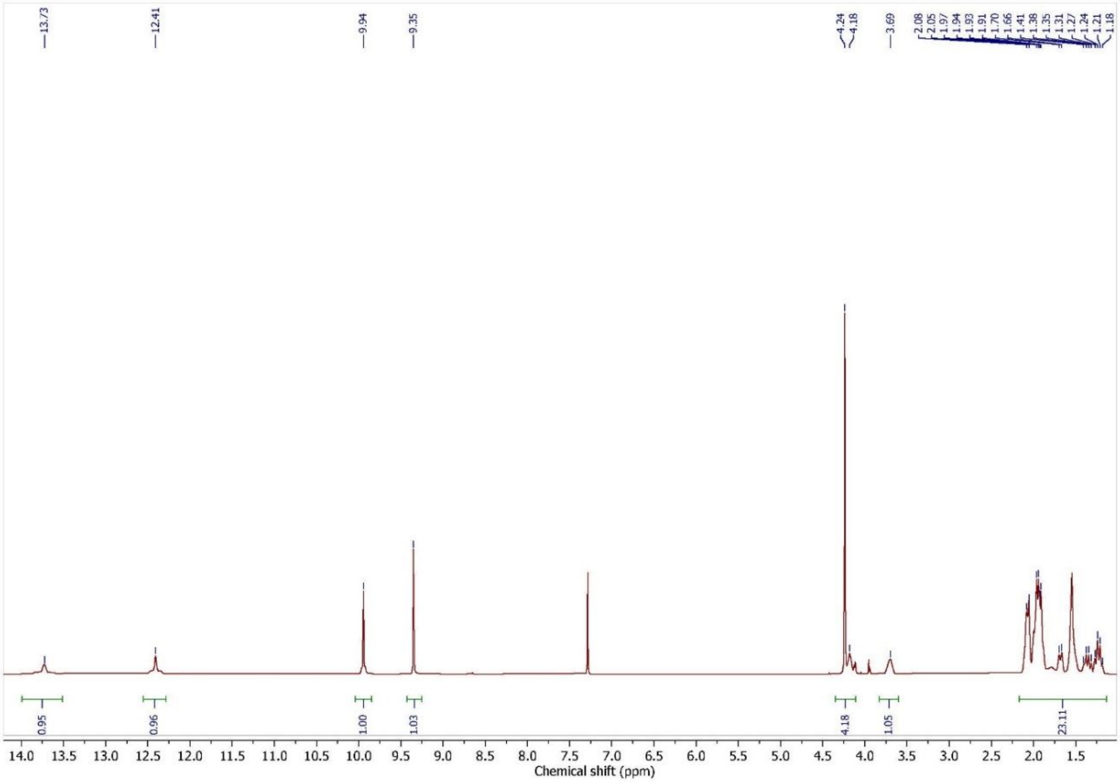

Figure S30. ^1H NMR spectra of **5** in $CDCl_3$ at RT.

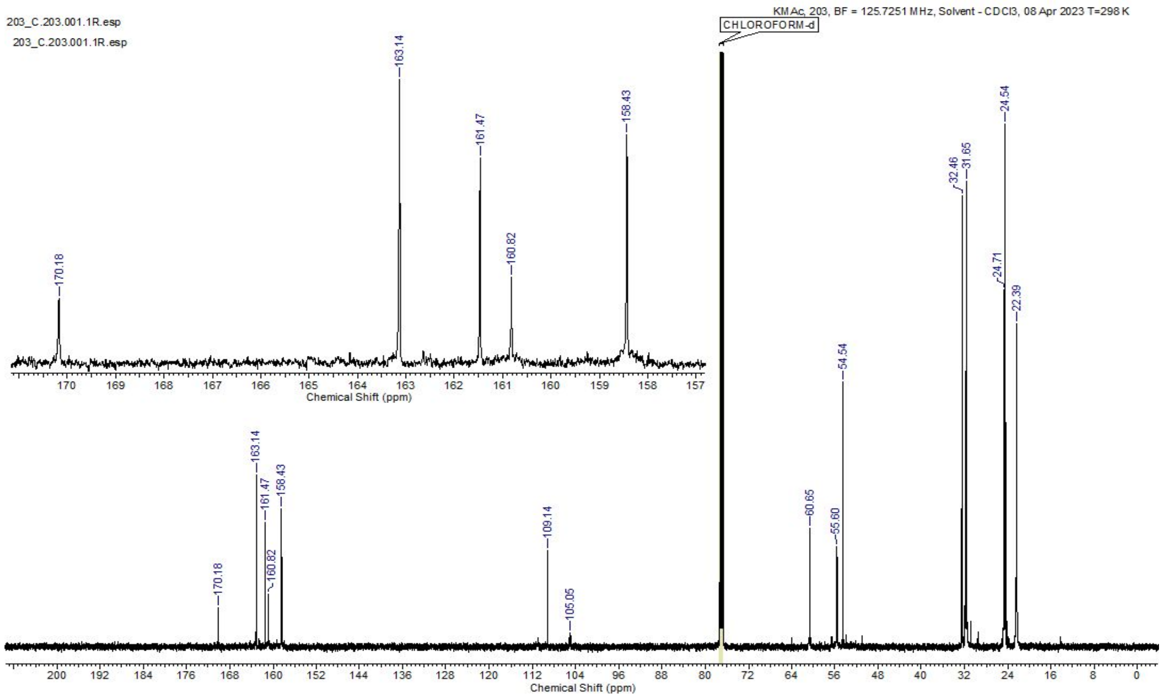

Figure S31. $^{13}C\{^1H\}$ NMR spectra of **5** in $CDCl_3$ at RT.

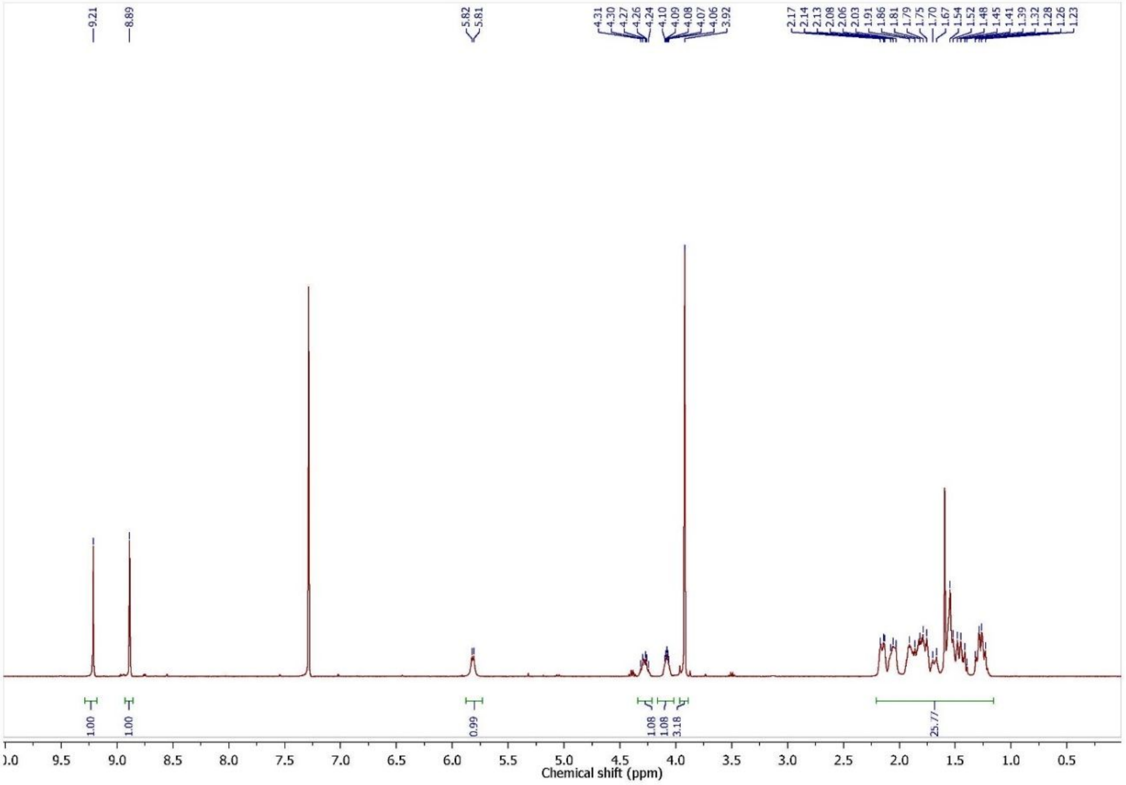

Figure S32. ^1H NMR spectra of **6** in $CDCl_3$ at RT.

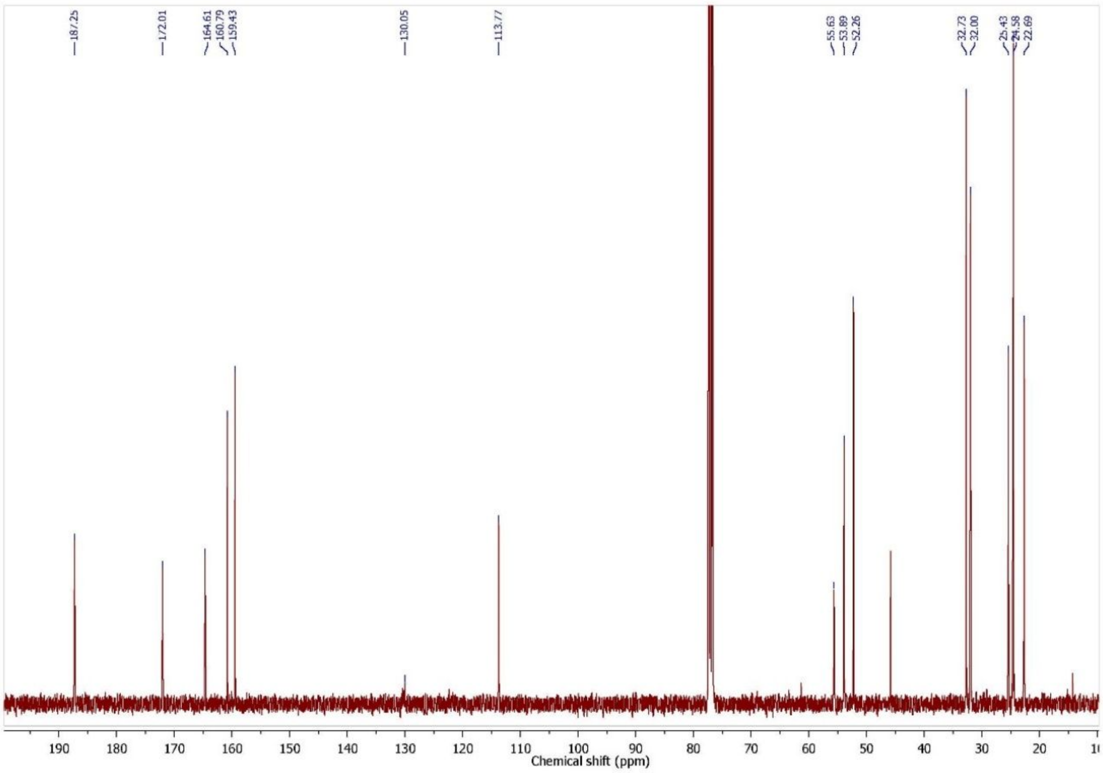

Figure S33. $^{13}C\{^{1}H\}$ NMR spectra of **6** in $CDCl_3$ at RT.

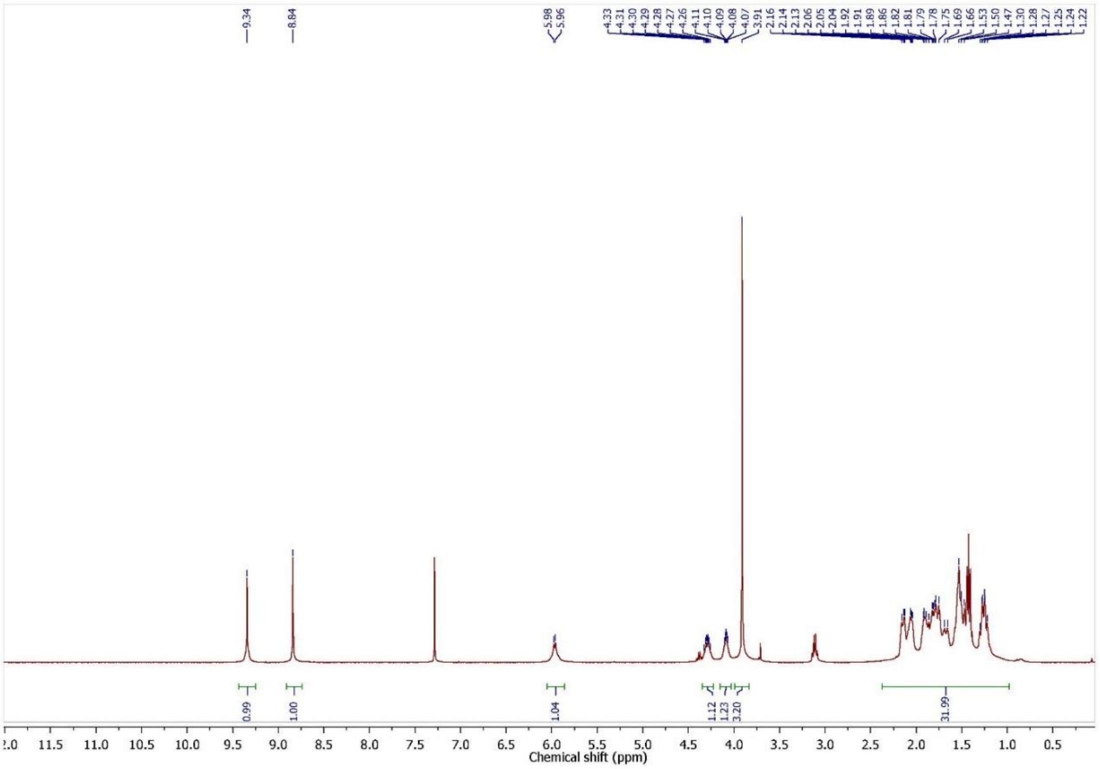

Figure S34. ^{1}H NMR spectra of **7** in $CDCl_3$ at RT.

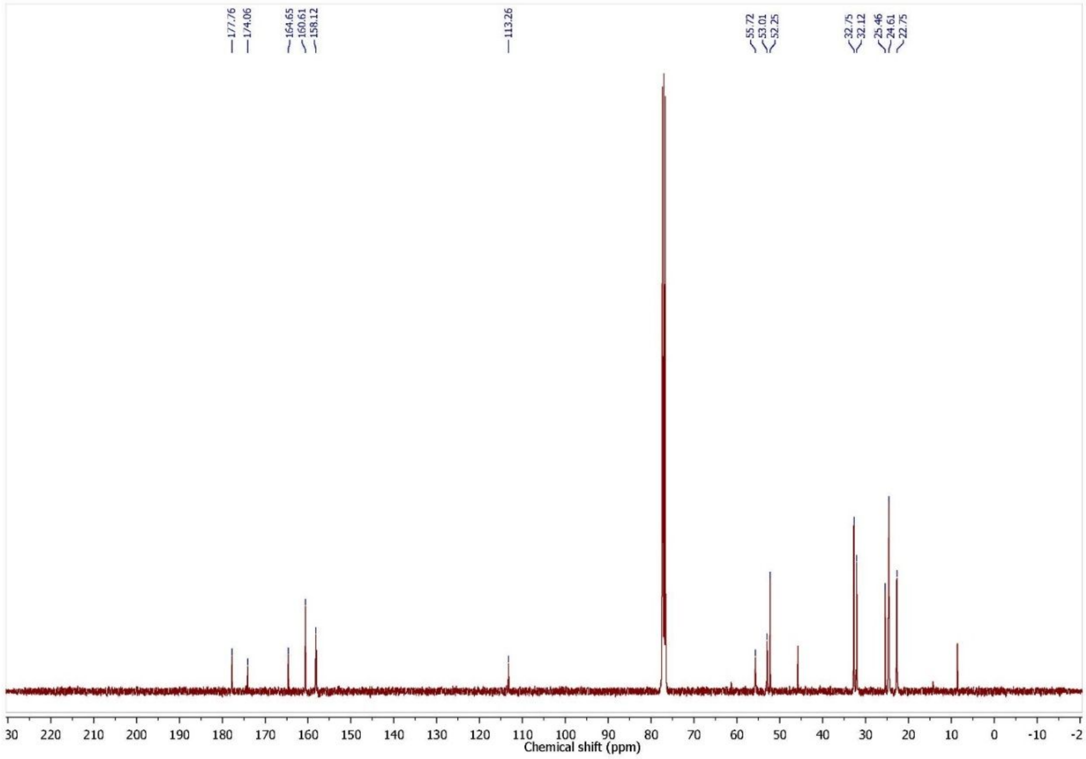

Figure S35. $^{13}C\{^{1}H\}$ NMR spectra of **7** in $CDCl_3$ at RT.

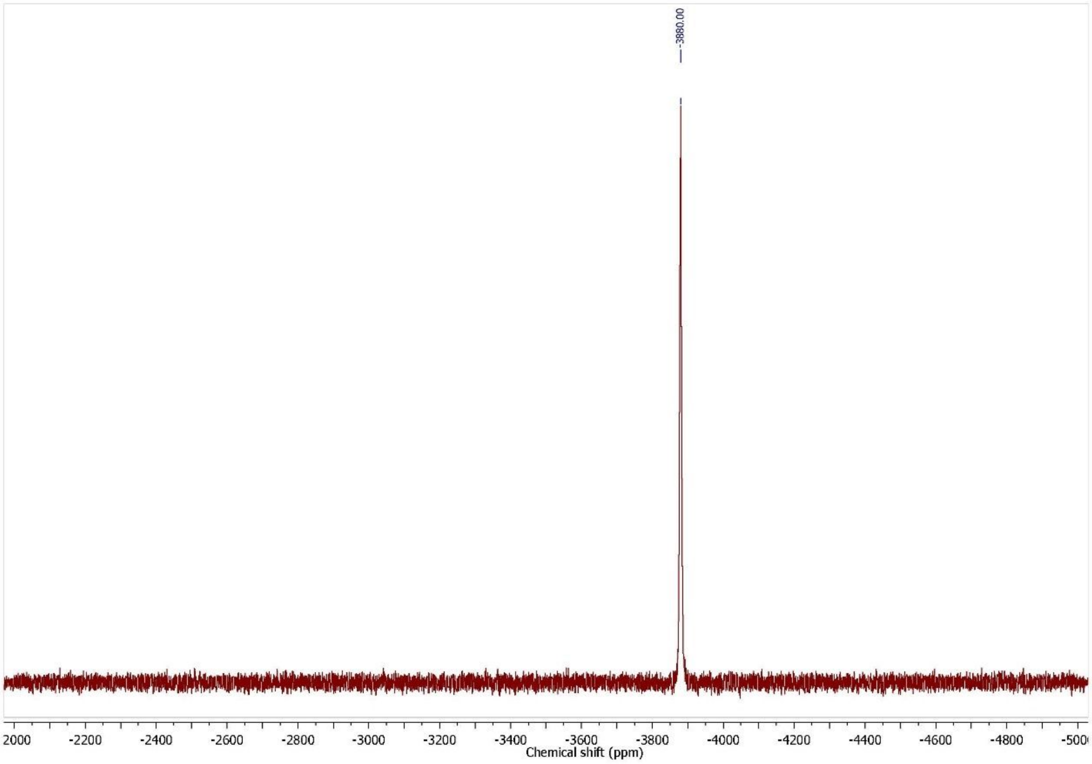

Figure S36. $^{195}Pt\{^{1}H\}$ NMR spectra of **7** in $CDCl_3$ at RT.

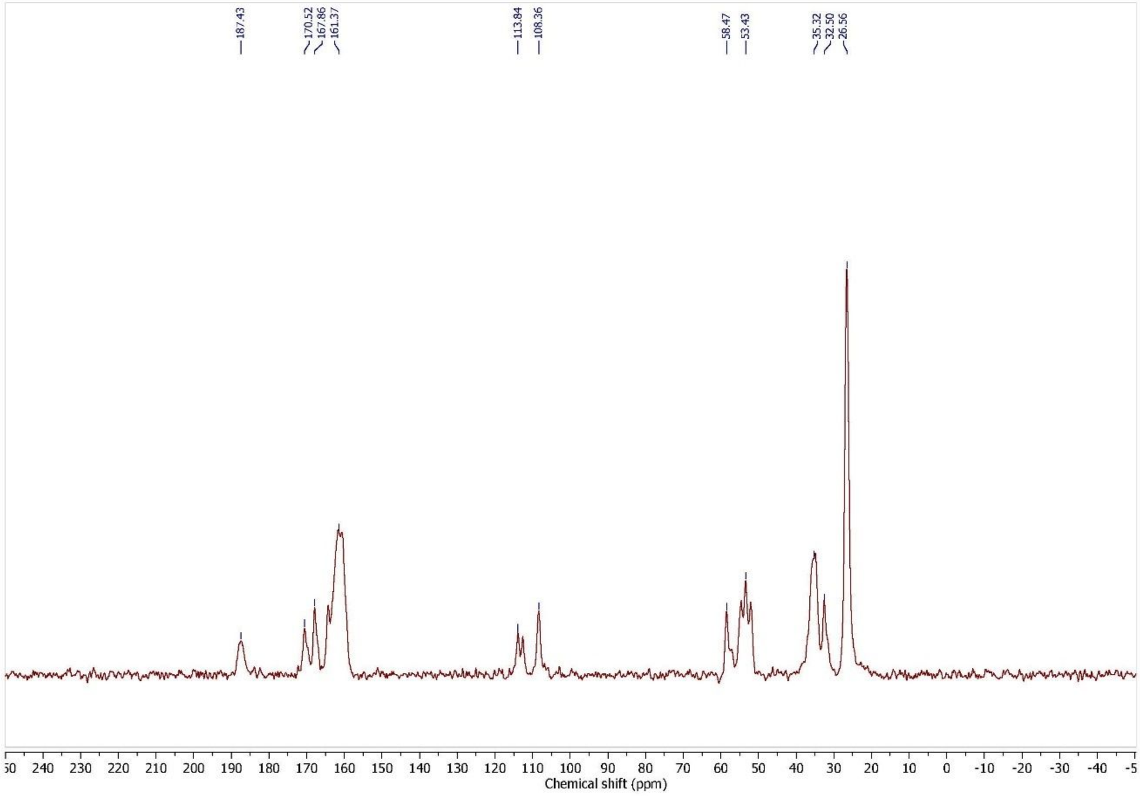

Figure S37. ^{13}C CP/MAS NMR spectra of **8** in solid stste at RT.

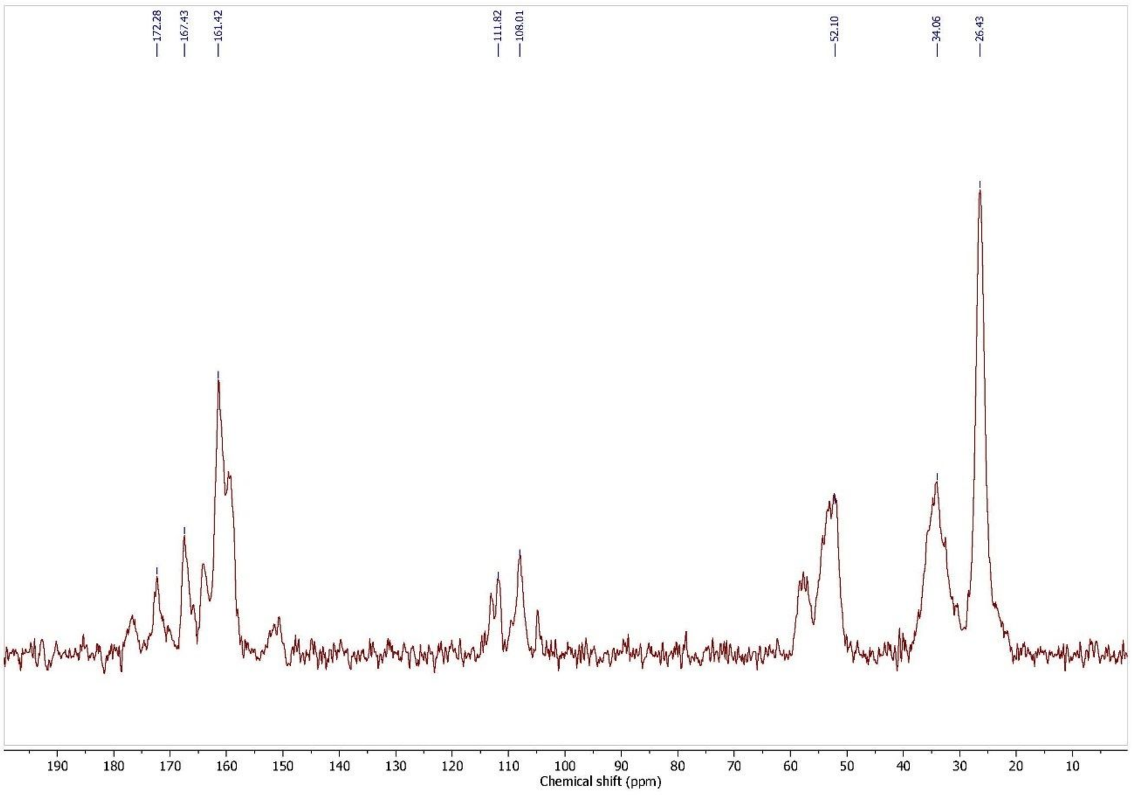

Figure S38. ^{13}C CP/MAS NMR spectra of **9** in solid state at RT.

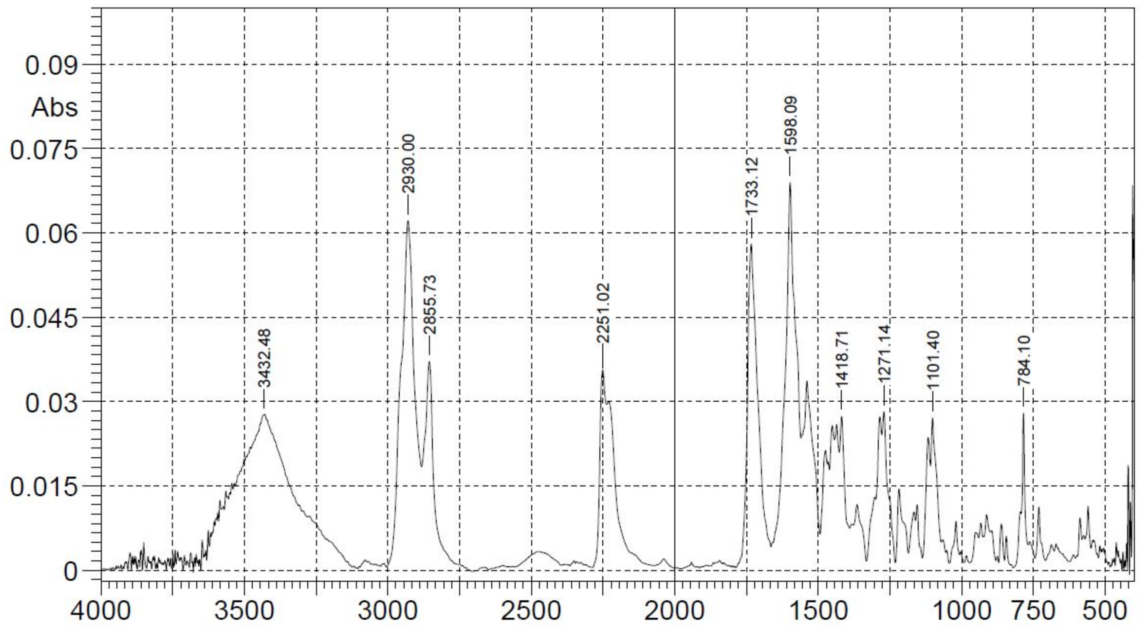

Figure S39. FTIR spectra of **5** in KBr pellets.

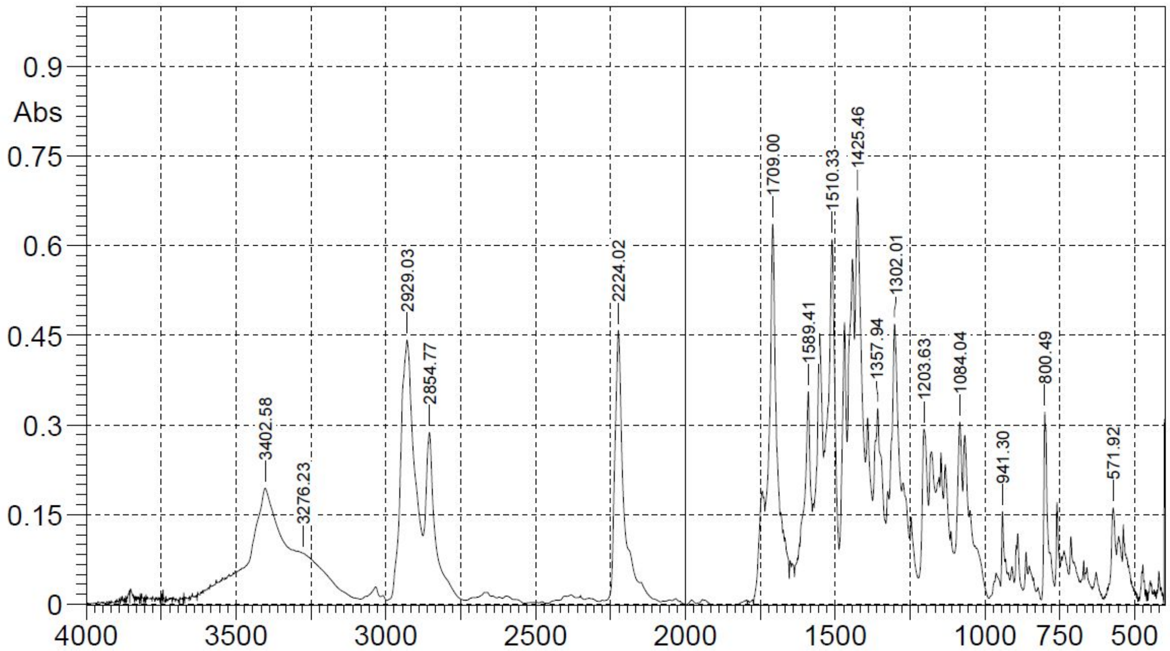

Figure S40. FTIR spectra of **6** in KBr pellets.

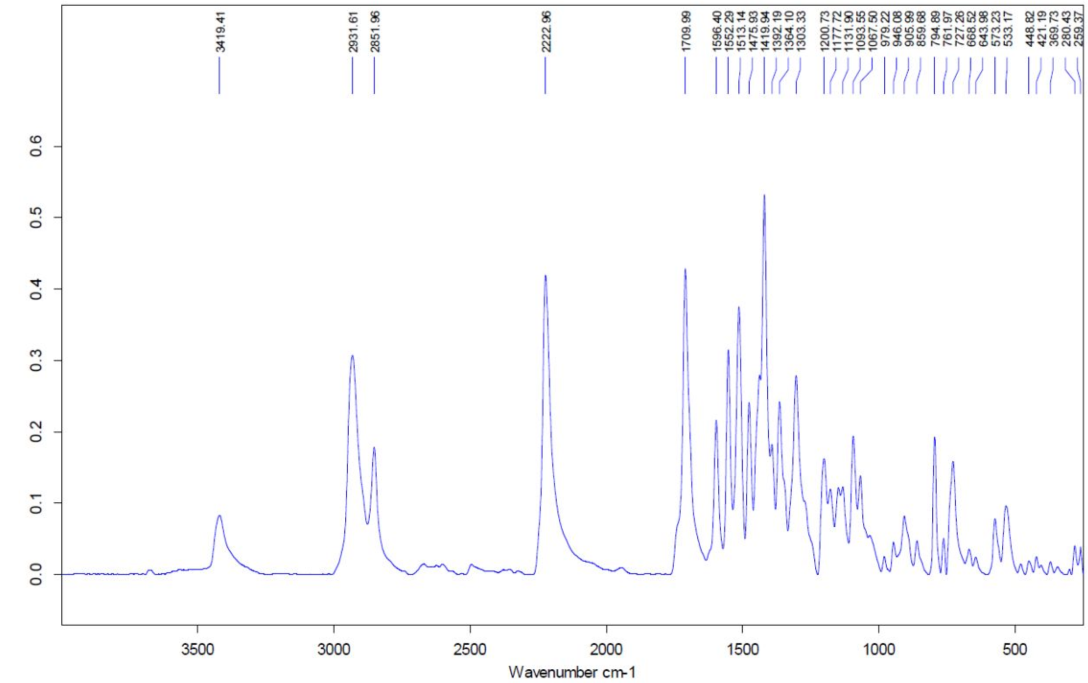

Figure S41. FTIR spectra of **7** in KBr pellets.

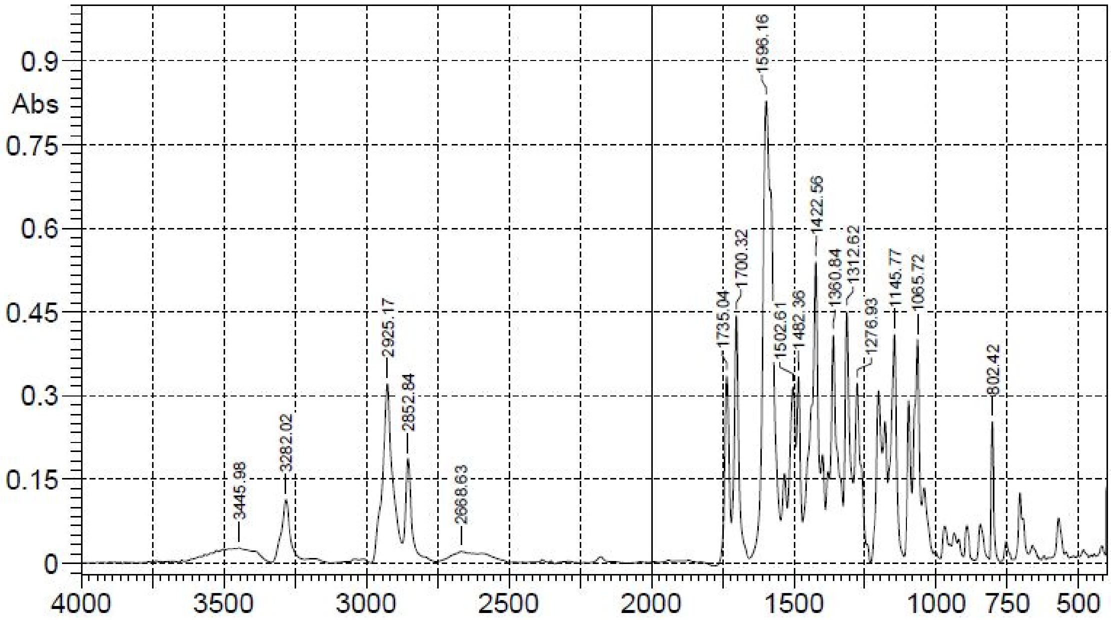

Figure S42. FTIR spectra of **8** in KBr pellets.

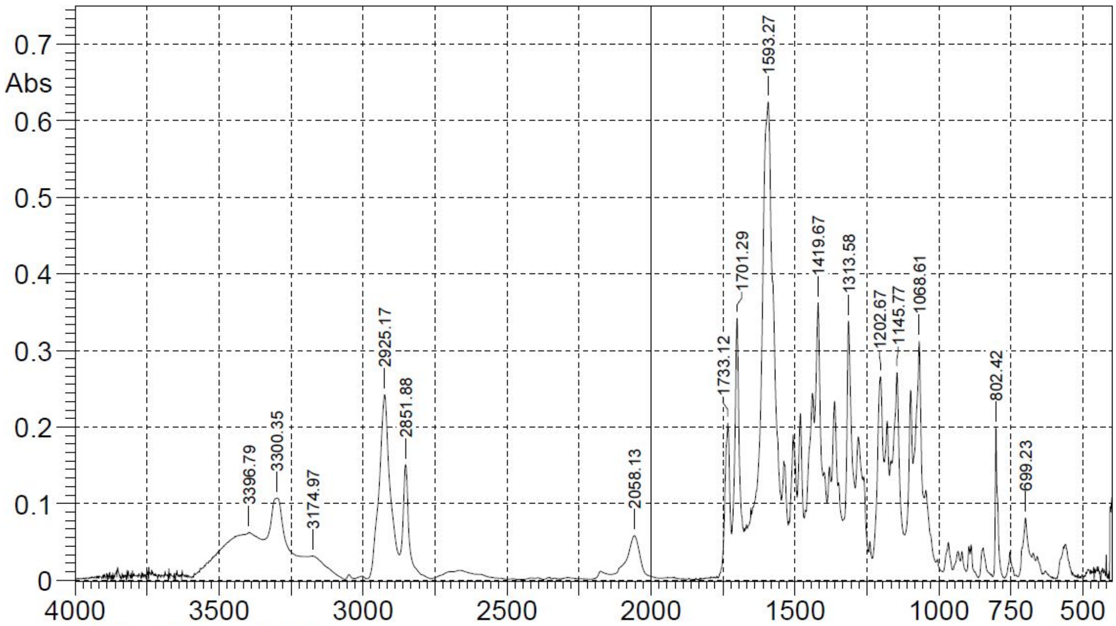

Figure S43. FTIR spectra of **9** in KBr pellets.

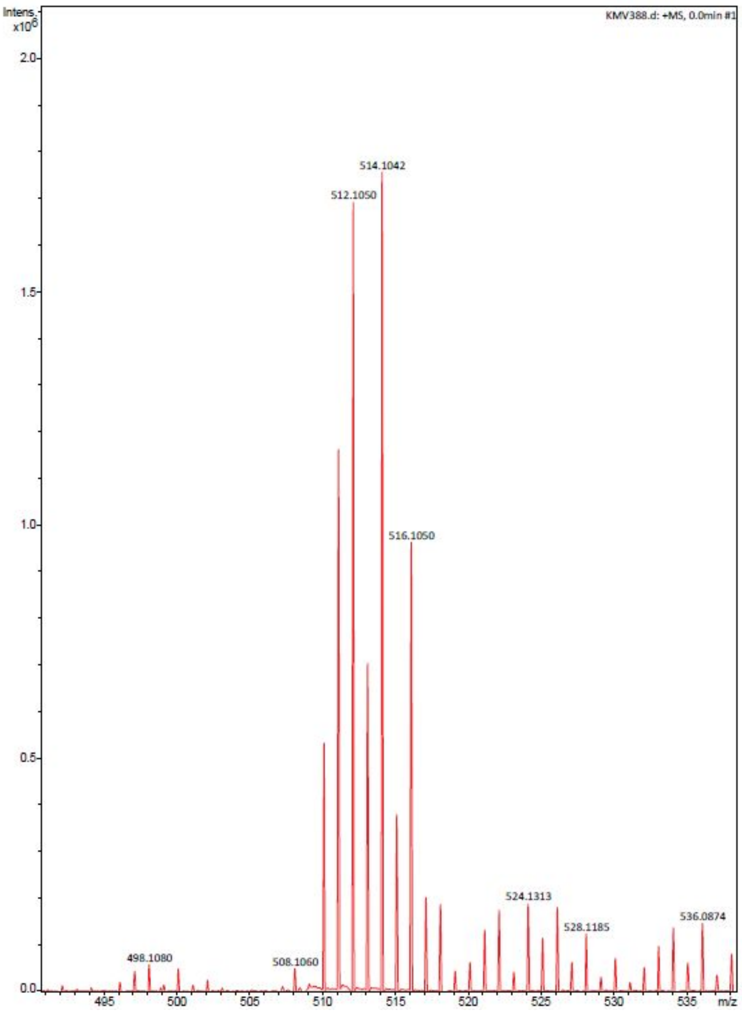

Figure S44. Mass spectra of $[M – Cl]^+$ for **4**.

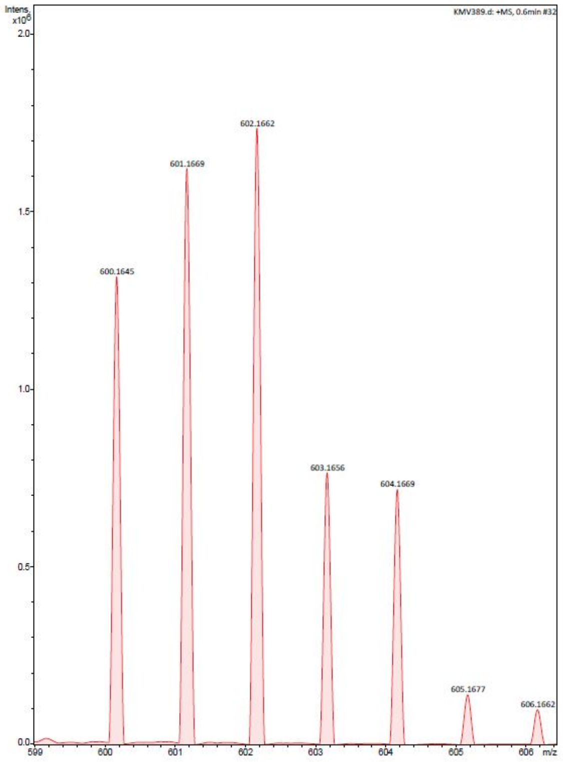

Figure S45. Mass spectra of $[M - Cl]^+$ for **5**.

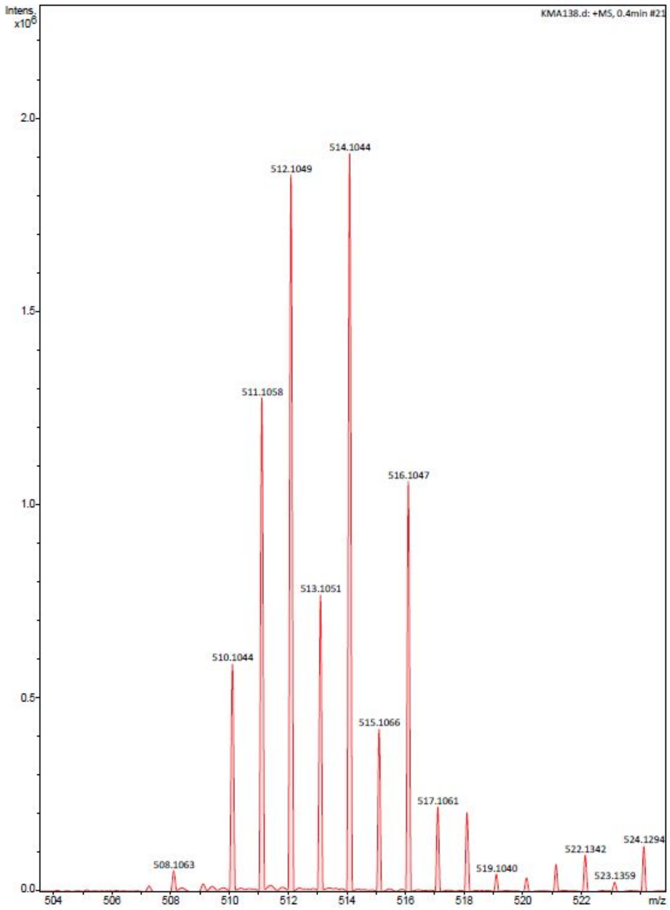

Figure S46. Mass spectra of $[M + H]^+$ for **6**.

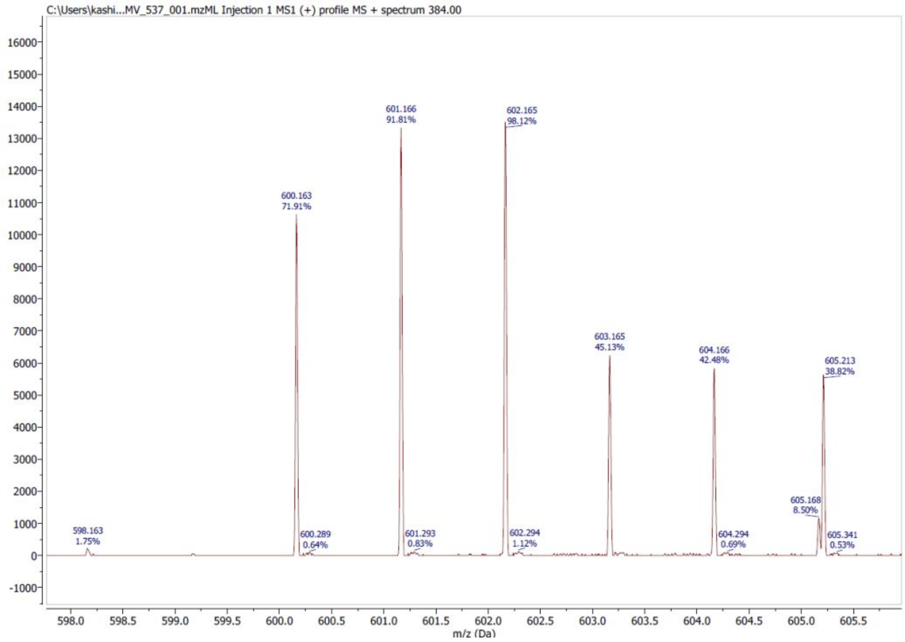

Figure S47. Mass spectra of $[M + H]^+$ for **7**.

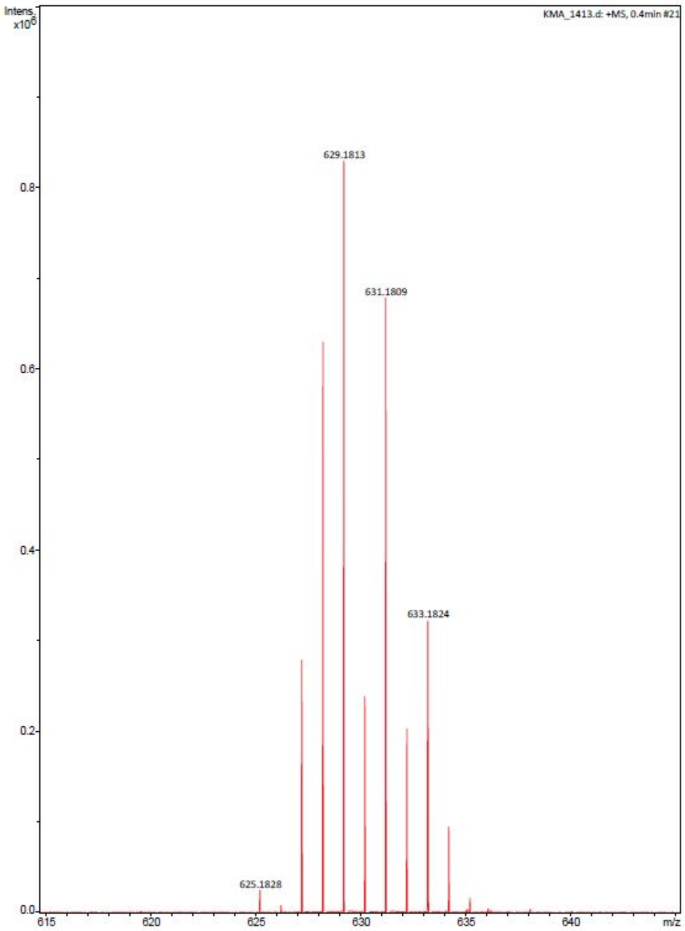

Figure S48. Mass spectra of $[M + H]^+$ for **8**.

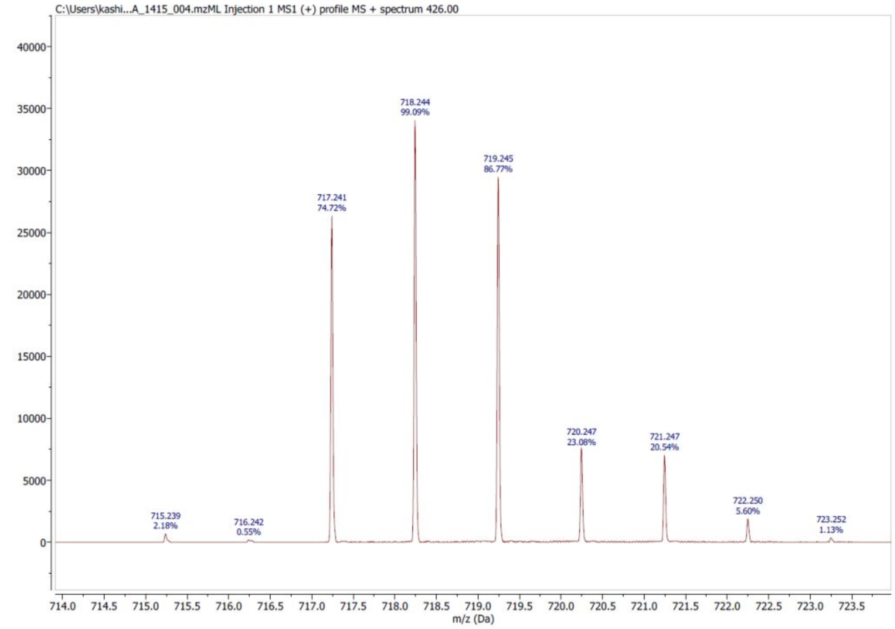

Figure S49. Mass spectra of $[M + H]^+$ for **9**.

References

1. Tskhovrebov, A. G.; Luzyanin, K. V.; Dolgushin, F. M.; Guedes da Silva, M. F. C.; Pombeiro, A. J. L.; Kukushkin, V. Y., Novel Reactivity Mode of Metal Diaminocarbenes: Palladium(II)-Mediated Coupling between Acyclic Diaminocarbenes and Isonitriles Leading to Dinuclear Species. *Organometallics* **2011,** *30* (12), 3362-3370.
2. Tskhovrebov, A. G.; Luzyanin, K. V.; Haukka, M.; Kukushkin, V. Y., Synthesis and Characterization of cis-$(RNC)_2Pt^{II}$ Species Useful as Synthons for Generation of Various (Aminocarbene)PtII Complexes. *J. Chem. Crystallogr.* **2012,** *42* (12), 1170-1175.
3. Kinzhalov, M. A.; Timofeeva, S. A.; Luzyanin, K. V.; Boyarskiy, V. P.; Yakimanskiy, A. A.; Haukka, M.; Kukushkin, V. Y., Palladium(II)-Mediated Addition of Benzenediamines to Isocyanides: Generation of Three Types of Diaminocarbene Ligands Depending on the Isomeric Structure of the Nucleophile. *Organometallics* **2016,** *35* (2), 218-228.
4. Martínez-Martínez, A.-J.; Chicote, M.-T.; Bautista, D.; Vicente, J., Synthesis of Palladium(II), -(III), and -(IV) Complexes with Acyclic Diaminocarbene Ligands. *Organometallics* **2012,** *31* (9), 3711-3719.
5. Boyarskaya, D. V.; Bulatov, E.; Boyarskaya, I. A.; Chulkova, T. G.; Rassadin, V. A.; Tolstopjatova, E. G.; Kolesnikov, I. E.; Avdontceva, M. S.; Panikorovskii, T. L.; Suslonov, V. V.; Haukka, M., Syntheses and Structures of a Series of Acyclic Diaminocarbene Palladium(II) Complexes Derived from 3,4-Diaryl-1H-pyrrol-2,5-diimines and Bisisocyanide Palladium(II) Complexes. *Organometallics* **2019,** *38* (2), 300-309.
6. Gee, J. C.; Fuller, B. A.; Lockett, H.-M.; Sedghi, G.; Robertson, C. M.; Luzyanin, K. V., Visible light accelerated hydrosilylation of alkynes using platinum–[acyclic diaminocarbene] photocatalysts. *Chem. Commun.* **2018,** *54* (68), 9450-9453.
7. Mikhaylov, V. N.; Sorokoumov, V. N.; Korvinson, K. A.; Novikov, A. S.; Balova, I. A., Synthesis and Simple Immobilization of Palladium(II) Acyclic Diaminocarbene Complexes on Polystyrene Support as Efficient Catalysts for Sonogashira and Suzuki–Miyaura Cross-Coupling. *Organometallics* **2016,** *35* (11), 1684-1697.
8. Sheldrick, G. M., A short history of SHELX. *Acta Crystallogr. A* **2008,** *64* (1), 112-122.
9. Dolomanov, O. V.; Bourhis, L. J.; Gildea, R. J.; Howard, J. A. K.; Puschmann, H., OLEX2: a complete structure solution, refinement and analysis program. *J. Appl. Crystallogr.* **2008,** *42* (2), 339-341.
10. Agilent, C. *CrysAlis PRO*, Agilent Technologies Ltd: Yarnton; Oxfordshire, 2014.
11. Kinzhalov, M. A.; Luzyanin, K. V.; Boyarskaya, I. A.; Starova, G. L.; Boyarskiy, V. P., Synthetic and structural investigation of $[PdBr_2(CNR)_2]$ (R =Cy, Xyl). *J. Mol. Struct.* **2014,** *1068*, 222–227.
12. Kinzhalov, M. A.; Boyarskii, V. P., Structure of isocyanide palladium(II) complexes and their reactivity toward nitrogen nucleophiles. *Russ. J. Gen. Chem.* **2015,** *85* (10), 2313-2333.
13. Crociani, B.; Boschi, T.; Belluco, U., Synthesis and reactivity of novel palladium(II)-isocyanide complexes. *Inorg. Chem.* **1970,** *9* (9), 2021-2025.
14. Jovanocić, B.; Manojlović-Muir, L.; Muir, K. W., Compounds containing platinum–carbon bonds. Part II. Crystal and molecular structure of cis-dichlorobis(phenyl isocyanide)platinum(II), cis-$[PtCl_2(CNPh)_2]$. *J. Chem. Soc., Dalton Trans.* **1972,** (11), 1178-1181.
15. Kinzhalov, M. A.; Kashina, M. V.; Mikherdov, A. S.; Katkova, S. A.; Suslonov, V. V., Synthesis of Platinum(II) Phoshine Isocyanide Complexes and Study of Their Stability in Isomerization and Ligand Disproportionation Reactions. *Russ. J. Gen. Chem.* **2018,** *88* (6), 1180-1187.
16. Hashmi, A. S. K.; Lothschütz, C.; Böhling, C.; Hengst, T.; Hubbert, C.; Rominger, F., Carbenes Made Easy: Formation of Unsymmetrically Substituted N-Heterocyclic Carbene Complexes of Palladium(II), Platinum(II) and Gold(I) from Coordinated Isonitriles and their Catalytic Activity. *Adv. Synth. Catal.* **2010,** *352* (17), 3001-3012.

17. Zou, C.; Lin, J.; Suo, S.; Xie, M.; Chang, X.; Lu, W., Palladium(II) N-heterocyclic allenylidene complexes with extended intercationic Pd···Pd interactions and MMLCT phosphorescence. *Chem. Commun.* **2018,** *54* (42), 5319-5322.
18. Hung, F. F.; Wu, S. X.; To, W. P.; Kwong, W. L.; Guan, X. G.; Lu, W.; Low, K. H.; Che, C. M., Palladium(II) Acetylide Complexes with Pincer-Type Ligands: Photophysical Properties, Intermolecular Interactions, and Photo-cytotoxicity. *Chem. Asian J.* **2017,** *12* (1), 145-158.
19. Gutierrez Suburu, M. E.; Maisuls, I.; Kösters, J.; Strassert, C. A., Room-temperature luminescence from Pd(II) and Pt(II) complexes: from mechanochromic crystals to flexible polymer matrices. *Dalton Trans.* **2022,** *51* (35), 13342-13350.
20. Theiss, T.; Buss, S.; Maisuls, I.; López-Arteaga, R.; Brünink, D.; Kösters, J.; Hepp, A.; Doltsinis, N. L.; Weiss, E. A.; Strassert, C. A., Room-Temperature Phosphorescence from Pd(II) and Pt(II) Complexes as Supramolecular Luminophores: The Role of Self-Assembly, Metal–Metal Interactions, Spin–Orbit Coupling, and Ligand-Field Splitting. *J. Am. Chem. Soc.* **2023,** *145* (7), 3937-3951.
21. Gangadharappa, S. C.; Maisuls, I.; Suburu, M. E. G.; Strassert, C. A., Enhanced phosphorescence of Pd(II) and Pt(II) complexes adsorbed onto Laponite for optical sensing of triplet molecular dioxygen in water. *Z. fur Naturforsch. - B J. Chem.* **2021,** *76* (10-12), 811-818.
22. Akaiwa, M.; Kanbara, T.; Fukumoto, H.; Yamamoto, T., Luminescent palladium complexes containing thioamide-based SCS pincer ligands. *J. Organomet. Chem.* **2005,** *690* (18), 4192-4196.
23. von der Stück, R.; Krause, M.; Brünink, D.; Buss, S.; Doltsinis, N. L.; Strassert, C. A.; Klein, A., Luminescent Pd(II) Complexes with Tridentate − Aryl-pyridine-(benzo)thiazole Ligands. *Z. Anorg. Allg. Chem.* **2022,** *648* (1), e202100278.
24. Turner, M. J.; McKinnon, J. J.; Wolff, S. K.; Grimwood, D. J.; Spackman, P. R.; Jayatilaka, D.; Spackman, M. A. *CrystalExplorer17*, University of Western Australia: Perth: Australia, 2017.
25. Spackman, P. R.; Turner, M. J.; McKinnon, J. J.; Wolff, S. K.; Grimwood, D. J.; Jayatilaka, D.; Spackman, M. A., CrystalExplorer: a program for Hirshfeld surface analysis, visualization and quantitative analysis of molecular crystals. *J. Appl. Crystallogr.* **2021,** *54* (3), 1006-1011.
26. McKinnon, J. J.; Jayatilaka, D.; Spackman, M. A., Towards quantitative analysis of intermolecular interactions with Hirshfeld surfaces. *Chem. Commun.* **2007,** (37), 3814–3816.
27. Bondi, A., Van der Waals Volumes and Radii. *J. Phys. Chem.* **1964,** *68* (3), 441–451.
28. Adamo, C.; Barone, V., Toward reliable density functional methods without adjustable parameters: The PBE0 model. *J. Chem. Phys.* **1999,** *110* (13), 6158-6170.
29. Grimme, S.; Jens, A.; Ehrlich, S.; Krieg, H., A consistent and accurate ab initio parametrization of density functional dispersion correction (DFT-D) for the 94 elements H-Pu. *J. Chem. Phys.* **2010,** *132* (15), 154104.
30. Frisch, M. J.; Trucks, G. W.; Schlegel, H. B.; Scuseria, G. E.; Robb, M. A.; Cheeseman, J. R.; Scalmani, G.; Barone, V.; Mennucci, B.; Petersson, G. A.; Nakatsuji, H.; Caricato, M.; Li, X.; Hratchian, H. P.; Izmaylov, A. F.; Bloino, J.; Zheng, G.; Sonnenberg, J. L.; Hada, M.; Ehara, M.; Toyota, K.; Fukuda, R.; Hasegawa, J.; Ishida, M.; Nakajima, T.; Honda, Y.; Kitao, O.; Nakai, H.; Vreven, T. *Gaussian 09, Revision B.01*, Gaussian, Inc.: Wallingford CT, 2010.
31. Bader, R. F. W., A quantum theory of molecular structure and its applications. *Chem. Rev.* **1991,** *91* (5), 893-928.
32. Savin, A.; Nesper, R.; Wengert, S.; Fässler, T. F., ELF: The Electron Localization Function. *Angew. Chem. Int. Ed.* **1997,** *36* (17), 1808-1832.
33. Contreras-García, J.; Johnson, E. R.; Keinan, S.; Chaudret, R.; Piquemal, J.-P.; Beratan, D. N.; Yang, W., NCIPLOT: A Program for Plotting Noncovalent Interaction Regions. *J. Chem. Theory Comp.* **2011,** *7* (3), 625-632.
34. Mayer, I.; Salvador, P., Overlap populations, bond orders and valences for 'fuzzy' atoms. *Chem. Phys. Lett.* **2004,** *383* (3), 368-375.
35. Glendening, E. D.; Landis, C. R.; Weinhold, F., Natural bond orbital methods. *Wiley Interdiscip. Rev. Comput. Mol. Sci.* **2012,** *2* (1), 1-42.

36. Lu, T.; Chen, F., Multiwfn: A multifunctional wavefunction analyzer. *J. Comput. Chem.* **2012,** *33* (5), 580-592.
37. Alvarez, S., A cartography of the van der Waals territories. *Dalton Trans.* **2013,** *42* (24), 8617-8617.
